# Pineapple Mycobiome Related to Fruitlet Core Rot Occurrence and the Influence of Fungal Species Dispersion Patterns

**DOI:** 10.3390/jof7030175

**Published:** 2021-02-28

**Authors:** Manon Vignassa, Jean-Christophe Meile, Frédéric Chiroleu, Christian Soria, Charlène Leneveu-Jenvrin, Sabine Schorr-Galindo, Marc Chillet

**Affiliations:** 1 Centre de coopération internationale en recherche agronomique pour le développement (CIRAD), UMR Qua-lisud, F-97410 Saint-Pierre, 97410 Réunion, France; meile@cirad.fr (J.-C.M.); christian.soria@cirad.fr (C.S.); marc.chillet@cirad.fr (M.C.); 2 Unité Mixte de Recherche Qualisud, Université de Montpellier, Avignon Université, Centre de Coopération Internationale en Recherche Agronomique pour le Développement, Institut Agro, Institut de Recherche pour le Développement, Université de La Réunion, Montpellier, France; sabine.galindo@umontpellier.fr; 3 Centre de coopération internationale en recherche agronomique pour le développement CIRAD, UMR PVBMT, 97410 Saint-Pierre, F-97410 La Réunion, France; frederic.chiroleu@cirad.fr; 4 Unité Mixte de Recherche Qualisud, Université de La Réunion, Centre de Coopération Internationale en Recherche Agronomique pour le Développement, Université de Montpellier, Institut Agro, Avignon Université, Sainte-Clotilde, France; charlene.leneveujenvrin@yahoo.fr

**Keywords:** *Fusarium*, pathogenic fungi, pineapple disease, *Talaromyces*

## Abstract

Fruitlet Core Rot (FCR) is a fungal disease that negatively impacts the quality of pineapple, in particular the ‘Queen Victoria’ cultivar. The main FCR causal agent has been identified as *Fusarium* *ananatum*. This study focused on the correlation between FCR disease occurrence, fungal diversity, and environmental factors. FCR incidence and fungal species repartition patterns were spatially contextualized with specific surrounding parameters of the experimental plots. The mycobiome composition of healthy and diseased fruitlets was compared in order to search for potential fungal markers. A total of 240 pineapple fruits were sampled, and 344 fungal isolates were identified as belonging to 49 species among 17 genera. FCR symptom distribution revealed a significant gradient that correlated to that of the most abundant fungal species. The association of wind direction and the position of proximal cultivated crops sharing pathogens constituted an elevated risk of FCR incidence. Five highly represented species were assayed by Koch’s postulates, and their pathogenicity was confirmed. These novel pathogens belonging to *Fusarium* *fujikuroi* and *Talaromyces* *purpureogenus* species complexes were identified, unravelling the complexity of the FCR pathosystem and the difficulty of apprehending the pathogenesis over the last several decades. This study revealed that FCR is an airborne disease characterized by a multi-partite pathosystem.

## 1. Introduction

*Ananas comosus* has become a highly appreciated fruit and is considered the best tropical fruit in terms of world exportation volumes, with 3.2 million tons in 2019 [1]. Pineapple production is affected by numerous pests and diseases, including Fruitlet Core Rot (FCR). The infection leads to brown discoloration of the flesh, impacting fruit acceptability by the consumers. FCR occurrence in the field of tropical and subtropical areas impacts pineapple post-harvest quality and both local and export markets. Current strategies focus on the creation of new cultivars trying to combine desired sensorial qualities and an optimal level of resistance to pathogens, as with the ‘MD-2’ cultivar [2]. Nevertheless, the ‘Queen Victoria’ cultivar still remains a favorite, especially for its flavor, in the Indian Ocean, as well as in Europe and some parts of Asia, despite its high susceptibility to a broad spectrum of pathogens [3]. Disease incidence and severity have increased in the producing regions, resulting in significant fruit quality depreciation and thus economic concerns [4]. The FCR pathosystem was originally considered as the relation between *Ananas comosus* var. *comosus* and the fungus *Fusarium verticillioides* (syn. *Fusarium moniliforme*) associated with *Penicillium funiculosum* [5,6,7]. However, the evolution of molecular tools led to the identification of *Fusarium ananatum* and *Talaromyces funiculosus* as causal agents of FCR in China, South Africa, Mauritius, and Reunion Island [4,8,9,10,11]. Contamination by soil-borne pathogens (such as *P. funiculosum*) occurs at pre-flowering stages, and the fungus remains latent in a blossom cup. During fruit development, causal agents spread into septal nectaries, but propagation is restricted by the lignification of young plant tissues. The accumulation of sugar following fruit ripening finally enables the fungus to counterbalance the plant defenses and then colonize host tissues [12]. These events trigger the browning of the flesh, whose expansion is limited to the fruitlet, without reaching the core or the skin of the pineapple fruit. Those symptoms are defined as ‘black spots’, thus constituting a typical marker of FCR-infected fruitlets. Nevertheless, the disease incidence could not be directly evaluated during pineapple cultivation or after harvesting due to the absence of external symptoms. The complexity of the FCR pathosystem relies on the presence of both diseased and healthy fruitlets within the same pineapple fruit, thus suggesting that pathogenesis could be driven by a specific microbiota. Thus far, the critical biotic parameters that mediate pineapple immunity or susceptibility remain unknown.

Plant diseases are closely related to numerous biotic and abiotic factors. Fungal species can be found in association with various plant species leading to a large-scale distribution and high levels of diversity according to their habitat [13,14,15]. Considering the wide range of pineapple crop systems and abiotic environments, fungi identified as belonging to *Trichoderma* spp. have been isolated in higher relative abundances in leaf and leaf sheath tissues when compared to roots and rhizospheric areas. By contrast, these plant sections are characterized by a microbiota, at least 70% of which is composed of bacterial species [16]. Nonetheless, there has been no study about fungal taxa richness and diversity associated with pineapple. Among abiotic factors, previous work has shown a link between FCR incidence and specific climatic conditions, which allowed for the elucidation of a predictive model based on a pluviothermic index. Predictions showed that, in subtropical regions (such as Reunion Island), the combination of a high altitude, high rainfall and cool temperatures (16 °C to 27 °C) led to an elevated risk of harvesting fruits with FCR symptoms [17,18]. In spite of this, the impact of environmental factors on the fruit mycobiome and pathogen dispersion patterns remains a ‘black box’ in the fate of this plant–pathogen interaction. Further studies on inoculum availability have reported the prevalence of disease severity related to the spatial distribution pattern on mango and banana crops [19,20]. Current ecological concerns have induced the development of alternative strategies for managing the spreading of fungal pathogens. To this end, multi-scale assays are conducted in order to fit with environmental changes and their impact on microbial epidemics [21,22]. The few studies dealing with short-distance dispersal patterns have mostly focused on vineyard [23], mango [19], and apple orchards [24]. Those data demonstrated the impact of soil and row management on conidia dispersion dynamics. One of the relevant aspects of estimating the infection pressure is the relation between the pineapple plot and its surrounding environment, notably the concomitant crops. Indeed, fungal isolates belonging to *Fusarium* genus are able to infect a broad spectrum of hosts such as fruits, small grain cereals, and horticultural plants [25,26,27].

Based on these assumptions, the main goal of the present study was to characterize the fungal flora of healthy and naturally infected pineapple fruitlets. From the data obtained, fungal species distribution was linked to the proximal environment of the pineapple plots. We also investigated the potential co-occurrence between fungal species as potential key factors for disease development. Thanks to a context-specific approach, we report here for the first time the contribution of five new pathogenic fungal species belonging to *Fusarium* and *Talaromyces* genera in FCR incidence in pineapple cv. ‘Queen Victoria’.

## 2. Materials and Methods

### 2.1. Plant Material and Agricultural Practices

**Figure 1:**
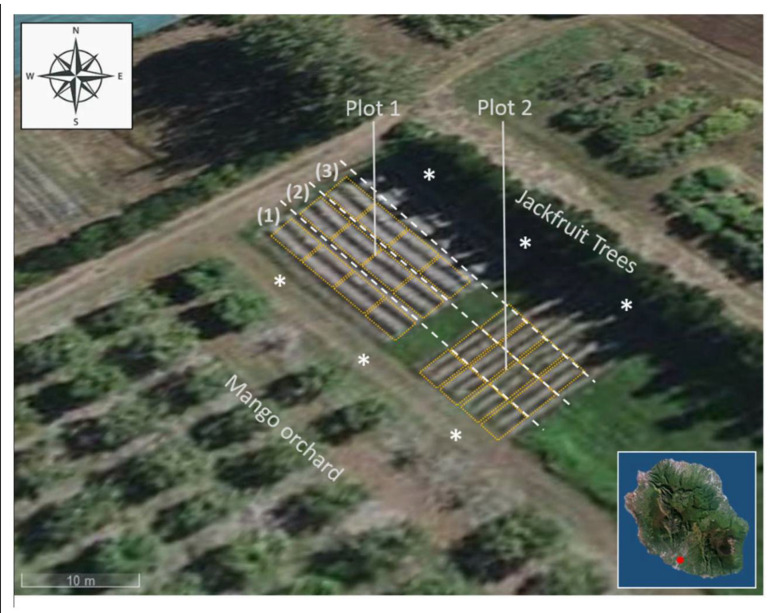
Environmental context of pineapple plots located in Reunion Island. Plot 1: North-West/South-East-directed rows; Plot 2: South-West/North-East-directed rows. (1) Plot sections proximal to the mango orchard, (2) central section of the plots, and (3) plot sections near the jackfruit trees. Quadrats are shown as yellow rectangles. White asterisks indicate coring points.

The experiment was conducted on the ‘Queen Victoria’ pineapple cultivar in accordance with the conventional cultural practices locally applied [28]. The plots were located in the southwest of Reunion Island at the Experimental Research Station of CIRAD Bassin-Plat, Saint-Pierre, located at an altitude of 150 meters above sea level (21°19′21′′ S, 55°29′26′′ E). The surface was divided into 2 plots with perpendicular row directions. Thereby, row orientations were North-West/South-East (N-W/S-E) and North-East/South-West (N-E/S-W) for Plot 1 and Plot 2, respectively (Figure 1). Each of the 2 experimental plots were subdivided into 3 sections (2 rows per section) along an N-E/S-W axis corresponding to rows adjacent to the mango orchard (1), central rows (2), and rows proximal to jackfruit trees (3). Each section was subdivided into 4 quadrats of 4 m long (*n* = 24). Meteorological data from flower induction treatment (FIT) in April 2018 to harvest in November 2018 were considered [29]. The wind data collection was performed by the Météo-France weather station located in Pierrefonds [30], Saint-Pierre, approximatively 6 km away from the plots (Table 1). A total of 240 fruits were harvested at the C1 stage, corresponding to 1/4 yellow fruit according to the shell’s color. The ripening was completed at 19 °C in a cold room until the C4 stage, when pineapples became entirely yellow.

### 2.2. FCR Symptom Occurrence and Fruitlets Sampling

Ten fruits per quadrat were collected. Each fruit was cut, and the number of black spots (infected fruitlets) was counted and recorded. FCR occurrence per fruit was then calculated for each section of each of the 2 plots. 

Fruitlets sampling was performed on 96 fruits randomly chosen (4 fruits per quadrat). For each pineapple fruit, 1 healthy-looking fruitlet (HF) and 1 naturally infected fruitlet (IF) were sampled with sterile equipment for a total of 192 fruitlets.

### 2.3. Isolation of Cultivatable Fungal Flora from Fruitlet and Soil Samples

#### 2.3.1. Fruitlet Tissues

The pineapple fruitlets collected (Section 2.2.) were deposited on Sabouraud glucose agar Petri dish (Biokar diagnostic, Solabia, Allonne, France) supplemented with 100 mg L^−1^ chloramphenicol at 27 °C in the dark. Fungal colony growth and isolation were processed daily following similar conditions until 5 days of incubation. Pure strains isolated were observed for morphological (colony aspect and color) and microscopic (macroconidia and microconidia) characterizations (Zeiss, Germany, model Axiostar Plus, magnification *x*100). These observations enabled the taxonomic assignation for each isolate at the genus level. Consequently, a conidia solution was prepared by adding 2 mL of SPW (Saline Peptone Water, Condalab, Torrejón de Ardoz, Madrid, Spain) to the Petri dish and gently scratching the mycelium surface with a sterile spreader. From the conidia solution, 1 mL was transferred into sterile cryovials with the addition of an equal volume of 40% glycerol (Sigma-Aldrich, Darmstadt, Germany) and stored at −80 °C. Another volume of 500 µL was recovered for the molecular identification procedure and stored at −20 °C.

#### 2.3.2. Soil

Soil sampling was conducted at 3 points 10 m apart on the 2 opposite junctions between the pineapple field and the surrounding crops: The jackfruit trees (North-East) and the mango orchard (South-West) (Figure 1). For each coring point (white asterisks), 1 g of a 5 cm soil layer was suspended in a sterile tube containing 9 mL of sterile physiological saline water with 9 g of NaCl supplemented with 1% Tween^®^ 80 (Merck, Darmstadt, Germany). Soil suspensions were then placed into a shaking incubator for 30 min at 25 °C and 200 rpm. Two technical repetitions per coring point were performed. For microbial assays, serial decimal dilutions were conducted to 10^−6^ in physiological saline water supplemented with 1% Tween^®^ 80. Fungi were isolated on PDA (Potato Dextrose Agar, BD Difco^TM^, Le Pont-de-Claix, France) supplemented with cycloheximide (10 mg L^−1^, Merck, Darmstadt, Germany) and on Sabouraud glucose agar supplemented with Chloramphenicol 100 mg L^−1^, after incubation for 5 days at 27 °C and 4 days at 30 °C, respectively. Fungal colonies were removed with a sterile scalpel and transferred into 1.5 mL sterile tubes with 500 µL of SPW. Isolates were finally stored at −20 °C prior to molecular procedures.

### 2.4. Molecular Characterization of Cultivatable Fungal Flora from Fruitlets and Soil Samples

According to the morphological analysis, genus-specific DNA primers were used to infer phylogenetic relationships between closely related genotypes of strains isolated from fruitlets. 

PCR amplification of fungal DNA was performed on conidia solutions with no DNA extraction procedure. For *Fusarium* sp. genus, the EF1α (TEF-1α) reference gene region was amplified by PCR. For *Aspergillus* sp., *Penicillium* sp., and *Talaromyces* sp. genus, the β-tubulin gene region was targeted for PCR amplification. Afterward, when no amplification could be obtained with those specific primers, an amplification of the Internal Transcribed Spacer (ITS) region was conducted with the ITS1F/ITS4 primer pair. The list of the primers used in this study is given in Table 2. The identification of soil fungal isolates was performed by PCR amplification of the Internal Transcribed Spacer (ITS) region.

**Table 2:** PCR primers sequences (forward and reverse) used for fungal strains identification.

Sequence ID of Primers Pair	Target Locus	Sequence Forward (5′→3′)	Sequence Reverse (5′→3′)	Product Length (bp)	References
ef1/ef2	Translation Elongation Factor-1α	ATGGGTAAGGAAGACAAGAC	GGAAGTACCAGTGATCATGTT	380–680	[31,32,33]
Bt2a/Bt2b	β-tubulin	GGTAACCAAATCGGTGCTGCTTTC	ACCCTCAGTGTAGTGACCCTTGGC	250–500	[34]
ITS1F/ITS2	Internal Transcribed Spacer 1	CTTGGTCATTTAGAGGAAGTAA	GCTGCGTTCTTCATCGATGC	145–695	[35,36]
ITS1F/ITS4	Entire Internal Transcribed Spacer		TCCTCCGCTTATTGATATGC	600–800	
GC-ITS1F/ITS4		CGCCCGCCGCGCGCGGCGGGCGGGGCGGGGGCACGGGGGGCTTGGTCATTTAGAGGAAGTAA	TCCTCCGCTTATTGATATGC		

PCR amplification reactions were performed in a final volume of 50 μL containing 0.3 μM of each primer, all the deoxyribonucleotide triphosphates (dNTPs) at 0.2 mM, 2 mM MgCl_2_, 10 μL of 5x GoTaq Flexi buffer, 1.25 U GoTaq^®^ G2 Flexi DNA polymerase (Promega, Charbonnières-les-Bains, France), and 2 μL of conidia solution. Conditions for the PCR amplification of the TEF-1α region were an initial denaturation at 95 °C for 3 min, 33 cycles at 95 °C for 30 s, 55 °C for 30 s, 72 °C for 1 min, and a final extension stage of 5 min at 72 °C. Similarly, PCR amplification reactions for the β-tubulin region were established as follows: An initial denaturation at 95 °C for 2 min, 30 cycles at 95 °C for 30 s, 62 °C for 30 s, and 72 °C for 30 s, and a final extension at 72 °C for 5 min. PCR amplification reactions for the ITS region were carried out as follows: An initial denaturation at 95 °C for 2 min, 40 cycles at 95 °C for 15 s, 53 °C for 30 s, and 72 °C for 45 s, and a final extension at 72 °C for 5 min. The PCR reactions were performed in a thermocycler (Veriti, Applied Biosystems, United Kingdom). PCR products were analyzed with the Qiaxcel^®^ Advanced System (Qiagen, Hilden, Germany) using size markers 250 bp - 4 kb.

The PCR products were sent to Macrogen (Amsterdam, The Netherlands) for purification and sequencing. The DNA sequences obtained were aligned with SnapGene v5.0 software, and identification was performed using a BLASTn similarity search. Sequences having a percentage of identity of at least 98% and 95% for fruit and soil isolates, respectively, and those with the lowest E-values were considered as belonging to the same species.

### 2.5. Koch’s Postulates

#### 2.5.1. Controlled Inoculations and Plant Material

Among fungal strains identified on infected fruitlets, 5 highly represented species were selected for pathogenicity assays. For each of the 5 species, 2–3 isolates were randomly tested according to the following correspondence: *Fusarium oxysporum* strains BP369 and BP460; *Fusarium proliferatum* strains BP114, BP429, and BP436; *Fusarium sacchari* strains BP138 and BP575; *Talaromyces stollii* strains BP054, BP185, and BP462; *Talaromyces amestolkiae* strains BP002, BP257, and BP605. First, strains of interest were grown on PDA for 7 days at 27 °C in the dark. For each isolate, an inoculum solution was prepared with sterile water as described in Section 2.3.1, and a final concentration was normalized at 10^5^ conidia per mL. Inoculations were performed on cv. ‘Queen Victoria’ pineapple fruits were harvested at the C1 stage and grown according to locally recommended conventional agricultural practices. For each of the 13 tested strains, 3 fruits were inoculated. For each fruit, 3 fruitlets were inoculated by injecting 25 µL of inoculum in a blossom cup from the upper, median, or basal fruits parts. Inoculations were also conducted with *Fusarium ananatum* strain BP383 as a positive control and with sterile water as a negative control (H_2_O), and a 2nd class of negative control fruits (Mock) was not inoculated for comparison to the initial fungal load of fruitlets. Fruits were then incubated for 7 days at 19 °C in a cold room. Thus, 16 different conditions (13 tested strains + 1 positive and 2 negative controls) were assayed on a total of 144 fruitlets from 48 pineapple fruits.

#### 2.5.2. Fruit Sampling

After incubation, inoculated fruitlets were sampled. The 3 infected or control fruitlets of each fruit were pooled and placed in a sealed sterile lab blender bag and mixed with 10 mL of SPW in a stomacher for 2 min at maximal speed. Prior to storage at −80 °C, 5 mL of lysate were recovered and supplemented with an equal volume of 40% glycerol.

#### 2.5.3. DNA Extraction

For each fruit sample and each pure fungal strain used for inoculation, 2 mL of lysate were collected in 2 mL sterile tubes and centrifuged at 14,000× *g* for 2 min. DNA extractions were performed on the biomass pellets with the FastDNA SPIN kit and the FastPrep-24 Instrument (MP Biomedicals^®^, llkirch, France) using Lysing Matrix A and Lysis Buffer CLS-Y in accordance with the manufacturer’s instructions.

#### 2.5.4. PCR-Denaturing Gradient Gel Electrophoresis (DGGE)

PCR amplifications were performed using the GC-ITS1F and ITS2 DNA primers (Table 2). A 40 bp GC-clamp was added to the 5’ end of the ITS1F primer in order to ensure that the DNA fragment remains partially double-stranded and that the region screened was in the lowest melting domain [37]. This method was limited by the fragment length, which should not exceed 500 bp. In spite of this, a large fragment length resulted in a poor gel resolution complicating the recovery of DNA bands. For these reasons, PCR-DGGE was performed on the ITS1 region (~200 bp), conferring a good resolution but discrimination of fungal strains only at genus level. PCR reaction was performed in a final volume of 50 μL containing 0.6 μM of each primer, all the deoxyribonucleotide triphosphates (dNTPs) at 200 μM, 2 mM of MgCl_2_, 10 μL of 5× GoTaq Flexi buffer, 1.25 U of GoTaq^®^ G2 Flexi DNA polymerase, and 1 μL of extracted DNA. PCR amplification reactions were carried out as follows: An initial denaturation at 95 °C for 2 min, 40 cycles at 95 °C for 15 s, 60 °C for 30 s, and 72 °C for 20 s, and a final extension at 72 °C for 5 min. The PCR reactions were performed in a thermocycler (Veriti, Applied Biosystems, United Kingdom). PCR products were then analyzed as previously described.

The PCR products were separated by DGGE using a Cleaver Scientific system (Cleaver Scientific, Rugby, Warwickshire, United Kingdom). Briefly, 30 µL of PCR amplicons were loaded onto 8% (*w/v*) polyacrylamide gels (acrylamide: N,N-methylene bis-acrylamide, 37.5:1, Sigma-Aldrich, Darmstadt, Germany) in a 1× TAE buffer (40 mM Tris-HCl pH 7.4, 20 mM sodium acetate, 1.0 mM Na_2_-EDTA). Electrophoresis was performed at 60 °C using a denaturing gradient ranging from 20% to 60% (100% denaturant corresponding to 7 M urea and 40% *v/v* formamide, Carlo Erba Reagents, Val-de-Reuil, France). The gels were run at 20 V for 10 min and then at 75 V for 16 h. After electrophoresis, the gels were stained for 1 h with ethidium bromide solution (50 μg mL^−1^ in 1× TAE), rinsed for 1 h in distilled water, and then visualized with a UV transilluminator (Bio-Rad^®^, Marnes-la-Coquette, France). Bands of interest were excised from the DGGE gels with a sterile scalpel as previously described [38]. Briefly, DNA of each band was then eluted in 100 µL of TE buffer (10 mM TrisHCl; 1 mM EDTA; pH 7.4, Sigma-Aldrich, Darmstadt, Germany) at 4 °C overnight. DNA was precipitated by adding 10 µL of sodium acetate (3 M, pH 5), 1 µL of glycogen (Molecular Grade, Roche Diagnostics, Meylan, France), and 300 µL of 100% ethanol and centrifuged at 15,000× *g* for 30 min at 4 °C. The supernatant was discarded, DNA pellets were washed with 500 µL of 70% ethanol, and, after 5 min of centrifugation, the DNA pellets were air-dried for 1 h. Finally, the DNA was re-suspended in 20 µL of TE buffer (10 mM TrisHCl; 1 mM EDTA; pH 7.4) and stored at −20 °C. PCR amplification reaction was performed on purified DNA with the ITS1F/ITS2 primer set. Reactions were carried out as follows: An initial denaturation at 95 °C for 2 min, 40 cycles at 95 °C for 15 s, 53 °C for 30 s, and 72 °C for 45 s, and a final extension at 72 °C for 5 min. PCR products were analyzed and sequenced as previously described in Section 2.4.

### 2.6. Computational Analysis

#### 2.6.1. Phylogenetic Analysis 

The determination of phylogenetic relationships between fungal strains was conducted by considering each genomic region. Multiple alignments of the nucleotide sequences were performed with the MEGAX computer program and the MUSCLE automatic alignments method [39,40]. Subsequently, the jModelTest v2.1.7 program was used to select the nucleotide substitution model for each sequence set [41]. The maximum-Likelihood analysis was inferred with the PhyML v3.0 program implemented with ‘ape’ R package according to the best fitting model [42,43]. Branch supports were tested with 100 bootstrap replications. Trees were then visualized and edited with the the FigTree v1.4.4 program.

#### 2.6.2. DGGE Band Pattern

Electrophoretic profiles were analyzed with CLIQS 1D Pro software (TotalLab, Newcastle-Upon-Tyne, United Kingdom). In the DGGE gel, for each sample (lane), the DNA band presence and its relative intensity were recorded. The Unweighted Pair-Group Method using Arithmetic Average (UPGMA) and Pearson coefficient correlation were used to build the dendrogram.

#### 2.6.3. Statistical Analysis

Data were analyzed with the R statistical language v4.0.3 (R Core Team, 2020). The effect of row direction and proximal crops on the mean number of infected-fruitlets per fruit was tested with a Deviance test on a generalized linear model with Poisson distribution, and a Tukey’s multiple comparison test. Principal Component Analysis (PCA) was computed with ‘FactoMineR’ on the centered-fungal species abundances with no scaling [44]. The 6 combinations between proximal crop and plot were considered as individuals. Results of PCA were then visualized with the ‘factoextra’ package [45]. Correlation between variables and each principal component of the factorial plane was tested at *p* < 0.05. Hierarchical clustering of species abundances in healthy and infected tissues was performed according to the Raup–Crick dissimilarity method from the ‘vegan’ package [46]. Species co-occurrence was computed with the ‘cooccur’ package based on a probabilistic model of calculations between expected and observed pairwise frequencies [47,48]. Correlation between species co-occurrence and fruitlet phenotype was assessed by a Mantel test on distance matrixes of each dataset.

## 3. Results

### 3.1. Fungal Flora of Healthy and Naturally Infected Fruitlets

**Figure 2:**
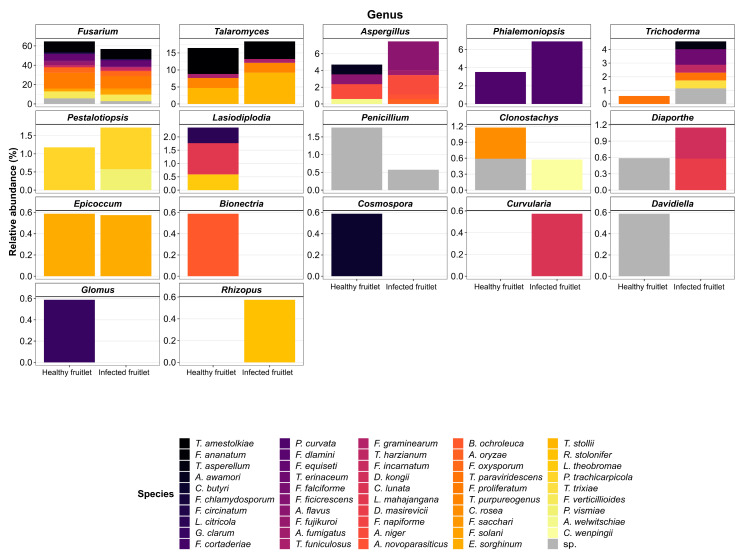
Relative abundances of fungal isolates sampled fro healthy and infected pineapple fruitlets at genus and species taxonomic levels.

The exploration of the mycobiome resulted in the identification of 170 isolates from healthy-looking fruitlets (HF), and 174 isolates from infected samples (IF) exhibiting black spots. In all, the 344 isolates belonged to 17 genera and represented a total of 49 different species. The relative abundance of the isolated fungal species identified five highly represented genera that corresponded to *Fusarium*, *Talaromyces*, *Aspergillus*, *Phialemoniopsis*, and *Trichoderma*, together supporting 90% (153 isolates) and 94% (164 isolates) of the cultivatable fungal flora of HF and IF, respectively (Table 3). The remaining isolates belonged to 12 genera (*Pestalotiopsis*, *Lasiodiplodia*, *Penicillium*, *Clonostachys*, *Diaporthe*, *Epicoccum*, *Bionectria*, *Cosmospora*, *Curvularia*, *Davidiella*, *Glomus*, and *Rhizopus*) with low relative abundances ranging from 0.58% to 2.35%. Although these were sparse isolates, *Lasiodiplodia*, *Bionectria*, *Cosmospora*, *Davidiella*, and *Glomus* were exclusively recorded in HF. Contrariwise, isolates corresponding to *Curvularia* and *Rhizopus* could only be observed in IF. The contextualization of predominant species among studied conditions revealed a decrease in relative abundances for *F. proliferatum* and *T. amestolkiae*, from 16.5% and 7.6% in HF to 12.6% and 5.2% in IF samples, respectively. By contrast, the other highly represented species were characterized by an increase in their relative abundances in the IF samples. This trend was notably observed for *T. stollii* and *P. curvata*, which extended from 4.7% and 3.5% in HF to 9.2% and 6.9% in IF, respectively (Figure 2). Particularly, only one *Trichoderma* species (*T. paraviridescens*) was isolated, showing a low relative abundance (0.6%) in HF, while this genus was supported by five species in IF (*T. asperellum*, *T. erinaceum*, *T. harzianum*, *T. paraviridescens*, and *T. trixiae*) contributing to 4.6% of the identified fungal flora. Although *F. ananatum* was the most frequently described FCR-associated pathogen [10,11,49], its occurrence and relative abundances were similar in HF and IF, with 11.2% and 10.3%, respectively. Interestingly, *F. proliferatum*, *F. ananatum*, *F. fujikuroi*, *F. circinatum*, *F. oxysporum*, *F. sacchari*, and *F. verticillioides* all belonged to the *Fusarium fujikuroi* species complex (FFSC) [49], which represented 43.5% and 37.9% of HF and IF mycobiomes, respectively. Similarly, the *Talaromyces purpureogenus* species complex was also highly represented following the identification of *T. stollii*, *T. amestolkiae*, and *T. purpureogenus*, together gathering 15.3% of isolates in HF and 17.2% in IF. Thus, those two species complexes together contributed to 58.8% and 55.2% of the cultivatable fungal flora of HF and IF, respectively.

### 3.2. Correlation between the Diversity of Fruitlet Mycobiomes and FCR Incidence

**Figure 3:**
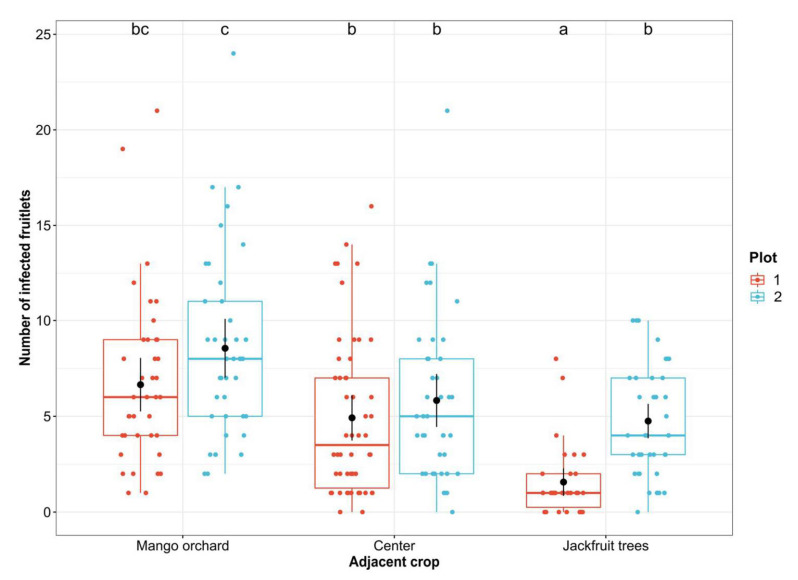
Scatter and box plots of numbers of FCR-infected fruitlets (IF) per pineapple fruit in the function of the adjacent crop according to plot orientation (Plot 1: Red, Plot 2: Blue). Black dots with bars show mean numbers of IF per pineapple with asymptotic confidence intervals at 95%. Letters indicate significant average differences between plot x sections (*n* = 40) according to Tukey’s multiple comparison test (*p* ≤ 0.013).

To determine the prevalence of FCR over the experimental plots, the number of IF per fruit was reported on a total of 240 pineapple fruits. The deviance test evidenced significant interaction between plot orientation and adjacent crops (*p* < 0.0001) on the average of IF per pineapple fruit. Considering Plot 1, the mean IF counts per pineapple fruit (mean ± sd) were 6.65 ± 4.36 for the row section proximal to the mango orchard, 4.92 ± 4.16 for rows localized in the center of the plot, and 1.57 ± 1.92 for rows near the jackfruits trees. Similar patterns were observed over rows of Plot 2, however, showing the highest infestation level with a mean of 8.55 ± 4.80 IF per pineapple fruit grown near the mango orchard, 5.82 ± 4.32 IF for pineapples in the plot center, and 4.75 ± 2.81 IF for fruits proximal to the jackfruit trees (Figure 3). Among Plot 1, the main significant differences in the average IF number were observed between mango orchard and jackfruit trees sections (*p* = 3.03 × 10^−7^) and between center and jackfruit trees sections (*p* = 5.88 × 10^−5^). Considering Plot 2, the average IF number per fruit were significantly different between mango orchard and jackfruit trees sections (*p* = 0.0002) and between center and mango orchard sections (*p* = 0.013). Moreover, the comparison data in jackfruit sections also evidenced a significant rise of the average IF number per fruit in Plot 2 compared to Plot 1 (*p* = 0.0001). 

**Figure 4:**
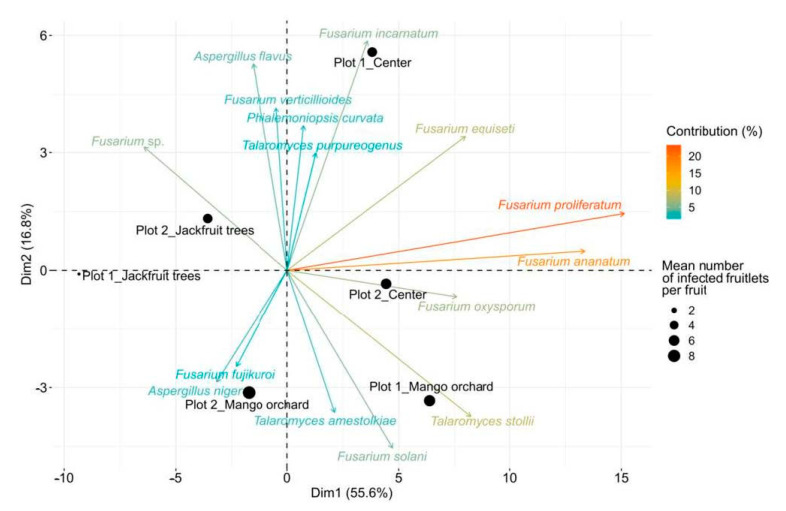
Principal component analysis (PCA) of combinations between proximal crop and plot direction. Only the 15 most contributory fungal species are shown (contributions ranging from 23.60% to 1.08%). Plot 1: North-West/South-East-directed rows; Plot 2: South-West/North-East-directed rows; mango orchard or jackfruit trees as adjacent crops, respectively; center: No adjacent crops. The size of each dot is proportional to the average number of infected fruitlets per fruit for each section of plots.

Considering the distribution profile of FCR symptoms, the distribution of the identified fungal species over the plots was investigated to search for a correlation. The first factorial axis (Dim1) of the PCA computed on the abundance of the 344 identified isolates according to plot sections explained 55.6% of the variance (Figure 4). Jackfruit trees and mango orchard sections of Plot 1 contributed to Dim1 with 48.9% and 22.8%, respectively. 

**Figure 5:**
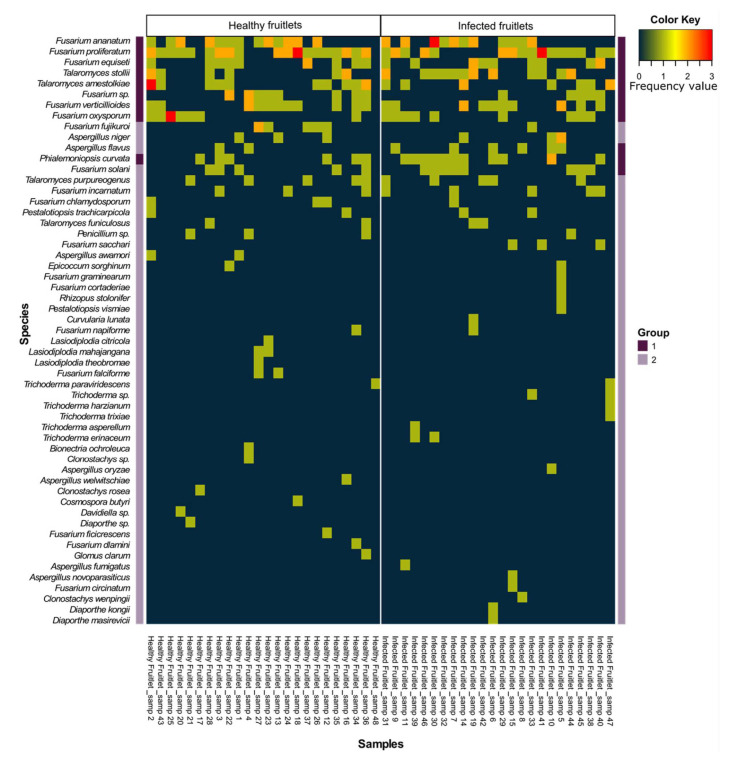
Heat map representation of fungal species frequencies in healthy and infected fruitlets of each quadrat. Group 1 corresponds to species identified in at least 25% of healthy and infected fruitlet sets, and Group 2 shows species identified in less than 25% of healthy and infected fruitlet sets.

Symmetrically, Dim1 was supported by *F. proliferatum*, *F. ananatum*, *T. stollii*, *F. equiseti*, and *F. oxysporum* with respective contributions of 30.4%, 23.7%, 9.02%, 8.4%, and 7.68%. Correlation between variables and the first component evidenced that *F. ananatum* (R = 0.99) and *F. proliferatum* (R = 0.96) were positively correlated to PC1 at *p* = 0.0002 and *p* = 0.002, respectively. Thus, the mango orchard section of Plot 1 was associated with elevated abundances of *F. proliferatum*, *F. ananatum*, *T. stollii*, *F. equiseti*, and *F. oxysporum* by opposition to the jackfruit trees section of the same plot. Additionally, 16.8% of the variance was explained by the Dim2. The construction of PCA factorial plane established that the center section of Plot 1 (57.71%) and mango orchard proximal rows of Plot 2 (18.1%) were the main contributive individuals of Dim2 in association with *F. incarnatum* (15.0%), *A. flavus* (12.2%), *F. solani* (9.0%), and *F. verticillioides* (7.5%). Moreover, *A. flavus* (R = 0.95), *F. incarnatum* (R = 0.84), *F. dlamini* (R = 0.83), *Diaporthe* sp. (R = 0.83), *D. masirevicii* (R = 0.83), *D. kongii* (R = 0.83), *C. butyri* (R = 0.83), *C. wenpingii* (R = 0.83), *A. fumigatus* (R = 0.83), and *F. verticillioides* (R = 0.82) were the main variables positively correlated to Dim2 at *p* ≤ 0.04. This demonstrates that center and mango orchard sections of Plot 1 were characterized by different structuration in fungal communities even if their average number of IF per fruit were not significantly different. In addition, the jackfruit trees section of Plot 1 that presented the lowest FCR incidence also demonstrated the poorest fungal diversity and abundances. Considering that the most contributive fungal species were also among the most abundant in fruitlet samples, this suggested that fungal species repartition was closely linked with FCR occurrence inside the plot sections. Consequently, relations between host tissues and fungal species prevalence were tested through the establishment of hierarchical clustering based on isolate counts in the 24 quadrats (Figure 5). Two distinct groups could be observed: Group 1 highlighted co-occurrence depicting similar profiles of highly represented fungal species, corresponding to identification in at least 25% of each sample set.

**Figure 6:**
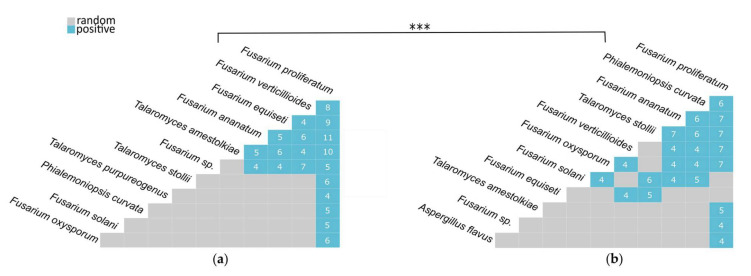
Matrixes of fungal species co-occurrences determined in healthy (**a**) and infected fruitlets (**b**) based on a pairwise approach. Significant positive associations are indicated in blue with the corresponding number of observations. Random associations are indicated in grey. The correlation between matrixes was assayed by the Mantel test (*** significance at *p* ≤ 0.001).

HF (*n* = 24) seems mainly characterized by *F. ananatum*, *F. proliferatum*, *F. equiseti*, *T. stollii*, *T. amestolkiae*, *F. verticillioides*, *F. oxysporum*, and *P. curvata*, together gathering 61.8% of the fungal flora. Interestingly, IF clustering (*n* = 24) shared the same species in addition to *A. flavus* and *F. solani*, which all contributed to 70.7% of the overall mycobiome diversity. In both types of fruitlet sets, a second group (Group 2) contained low abundance species identified in a limited number of quadrats and were thus considered as non-prevalent species for the rest of the analyses. Fungal communities were thus explored according to a combinatorial approach. Pairwise effect sizes were computed and revealed positive co-occurrence patterns in both HF and IF (only significant species combinations were displayed). Both matrixes showed 19 and 22 positive pair profiles for HF and IF fungal species datasets, respectively (Figure 6). Positive associations evidenced species pairs with significantly greater and large frequency than predicted by the model. By contrast, random associations were defined by observed values nearly equal to expectations and with no significant difference.

*F. proliferatum* was found in a positive combination with all individuals belonging to Group 1. Despite being the most prevalent species in the dataset, *F. proliferatum* had the highest number of pairwise associations in both tissues by positively co-occurring with 10 species (69 observations) in HF and with 8 species in IF (47 observations). Alternatively, co-occurrence patterns of several species were contrasted by presenting fewer combinations in IF samples than in HF samples. This trend was reported for *F. equiseti* and *T. amestolkiae*, which were represented 28 and 25 times in associations with 5 and 4 species in HF but only combined 9 and 5 times with 2 and 1 species in IF, respectively. On the contrary, 5 species displayed more positive combinations in the IF set than in the HF set. As an example, *P. curvata* was described to be positively associated only with *F. proliferatum* in HF (5 observations), while a combination with 6 species (31 observations) was highlighted in IF samples. This profile was also observed, to a lesser extent, for *T. stollii* (HF: 1 association observed 6 times; IF: 5 associations observed 31 times), *F. solani* (HF: 1 association observed 5 times; IF: 4 associations observed 19 times), and *F. oxysporum* (HF: 1 association observed 6 times; IF: 5 associations observed 23 times). Association patterns suggested that the fungal flora associated with HF and IF was structured. However, the Mantel test processed on distance matrixes demonstrated a significant relationship between the mycobiomes of the HF and IF samples (*p* = 0.0001). This revealed that species associations did not depend on the fruitlet phenotype (healthy or infected) when considered through a pairwise approach. 

**Figure 7:**
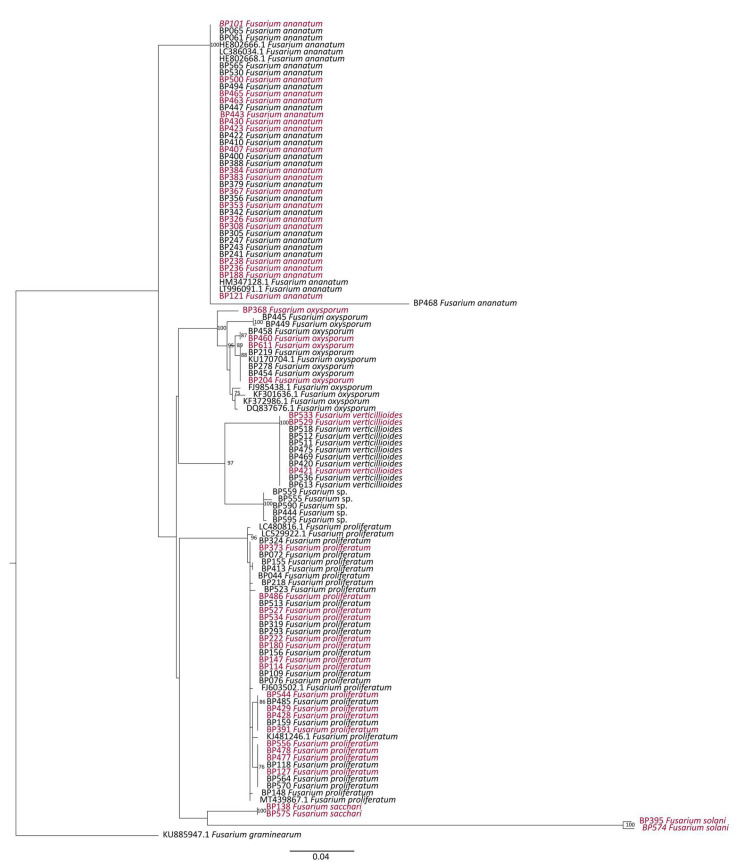
Phylogenetic tree of *Fusarium* species computed by Maximum Likelihood (ML) analysis (TIM2ef+G as the best fit model) and based on a TEF-1α sequences dataset. Only ML bootstrap branches that support values greater than 70% are shown. Strains isolated from infected (IF) and healthy fruitlets (HF) are indicated in red and black, respectively. The tree is rooted with *F. graminearum* accession KU885947.1.

Among plants pathogenic species, strains may exhibit a contrasting level of virulence, partly due to a genetic polymorphism inherited from co-evolution. TEF-1α and ITS gene sequence datasets represented, respectively, 101 and 98 *Fusarium* DNA sequences, with at least 98% of identity according to the reference nucleotide sequence from NCBI (Table S1). These loci were submitted to phylogenetic analyses following the Maximum Likelihood (ML) approach, with a bootstrap of 100 replications. Over the TEF-1α region, 6 clades related to the 6 different species represented in this sequence dataset were discriminated without the distinction of strains isolated from HF or IF samples, thus describing a high level of homology (Figure 7).

**Figure 8:**
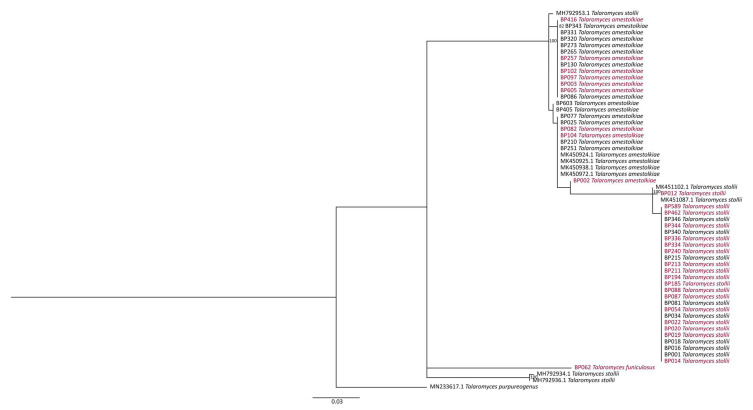
Phylogenetic tree of *Talaromyces* species computed by Maximum Likelihood (ML) analysis (TPM2+G as the best fit model) and based on a β–tubulin sequences dataset. Only ML bootstrap branches that support values greater than 70% are shown. Strains isolated from infected (IF) and healthy fruitlets (HF) are indicated in red and black, respectively. The tree is rooted with *T. purpureogenus* accession MN233617.1.

The same method was performed over the 61 *Talaromyces* strains. All of the 48 isolates characterized under the β-tubulin region corresponded to *T. amestolkiae* and *T. stollii*. As for the *Fusarium* strains, 2 clades were distinguished in concordance with the represented species (Figure 8). Strains were closely related inside clades, regardless of the phenotype (infected or healthy) of the fruitlets. This indicated that species virulence and pathogenicity were not linked to a polymorphism of TEF-1 α, ITS, or β-tubulin regions. Moreover, sequence analysis on TEF-1α and β-tubulin loci enabled a higher taxonomic resolution when compared to ITS (Figures S1 and S2).

### 3.3. Potential Sources of Inoculum and Dispersion Patterns

**Figure 9:**
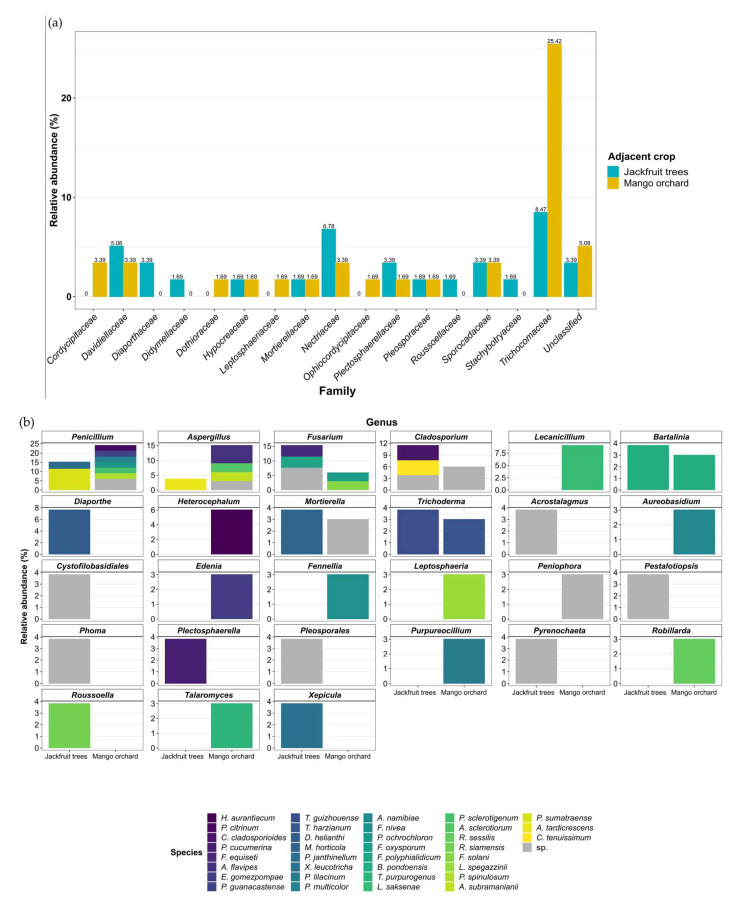
Relative abundances of fungal isolates identified from soil sampled on either side of the pineapple plots (jackfruit trees or mango orchard) at (**a**) family, (**b**) genus, and species taxonomic levels.

The distribution of fungal species over the experimental plots suggested the presence of an important inoculum source. Fungal flora of the soil at the interface between pineapple and jackfruit trees or the mango orchard was studied in order to elucidate its origin. Along the two junctions, 26 (mango orchard) and 33 (jackfruit trees) isolates were identified as belonging to 27 genera and 35 species. Most of the identified species belonged to the Trichocomaceae (32.2%) and Nectriaceae (10.2%) families (Figure 9a). The Trichocomaceae family was represented by *Talaromyces* (teleomorph of *Penicillium*), *Penicillium*, and *Aspergillus* species, which were detected in lower abundances in the soil related to jackfruit trees (19.2%) than in the mango orchard soil (45.6%), respectively.

Talaromyces was strictly represented by T. purpureogenus, while 8 Penicillium species were isolated: P. janthinellum, P. sumatraense, P. citrinum, P. guanacastense, P. multicolor, P. ochrochloron, P. sclerotigenum, and P. spinulosum. This family was also supported by 4 Aspergillus species: A. sclerotiorum, A. subramanianii, A. flavipes, and A. tardicrescens. Moreover, the Nectriaceae family was only represented by Fusarium species. The fungal isolates that belonged to Fusarium genus were detected at higher frequencies close to the jackfruit trees (15.4%) when compared to the mango orchard interface (6.1%) (Figure 9b, Table S2). Considering the diversity, two distinct species were identified in each junction: F. equiseti/F. polyphialidicum at the jackfruit trees and F. oxysporum/F. solani at the mango orchard interface. Remarkably, among all fungal soil isolates, no F. ananatum was identified. Subsequent isolates belonged to the Cladosporium, Plectosphaerella, Trichoderma, Diaporthe, Mortierella, Xepicula, Bartalinia, Roussoella, Acrostalagmus, Cystofilobasidiales, Pestalotiopsis, Phoma, Pleosporales, Pyrenochaeta, Heterocephalum, Edenia, Purpureocillium, Aureobasidium, Fennellia, Lecanicillium, Robillarda, Peniophora, and Leptosphaeria genera and were found with relative abundances ranging from 3% to 11.5%. Several contrasted profiles were noticed with the strict characterization of Lecanicillium, Heterocephalum, Aureobasidium, Edenia, Fennellia, Leptosphaeria, Peniophora, Purpureocillium, Robillarda, and Talaromyces genera in samples from the soil interface with the mango orchard. In contrast, Diaporthe, Acrostalagmus, Cystofilobasidiales, Pestalotiopsis, Phoma, Plectosphaerella, Pleosporales, Pyrenochaeta, Roussoella, and Xepicula genera were exclusively detected in the jackfruit tree junctions.

To evaluate the influence of abiotic factors on the fungal species spreading, meteorological parameters were obtained for the seven-month period from the flower induction treatment to harvest. Average temperatures ranged from 19.5 °C to 23.49 °C, with a low average rainfall below 5 mm. Data also demonstrated that the major wind direction was south-east during anthesis and fruit development stages, with a gust speed between 69 and 88 km h^−1^ (Table 1). The dynamics of these factors typically described the setting of the austral winter.

### 3.4. Koch’s Postulates for the Determination of New FCR Pathogens

In IF samples, species belonging to the *Fusarium fujikuroi* species complex were represented with high frequencies and concomitantly with two *Talaromyces* species related to the *Talaromyces purpureogenus* species complex. Koch’s postulates were verified for all of the following species: *F. proliferatum*, *F. sacchari*, *F. oxysporum*, *T. stollii*, and *T. amestolkiae*, in order to test for their pathogenicity. 

**Figure 10:**
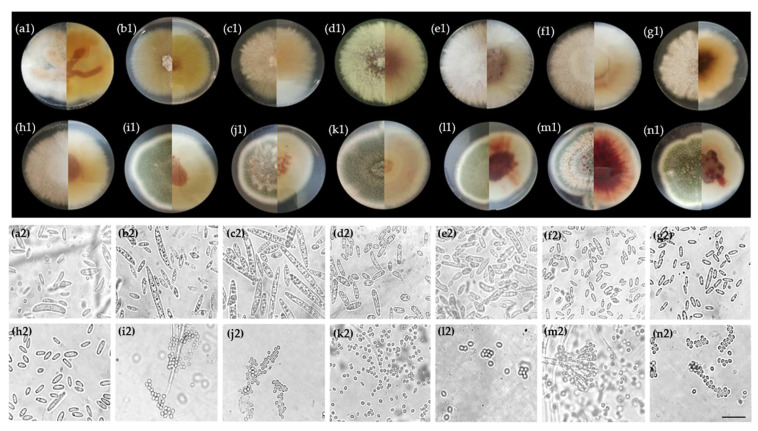
Morphological observations of fungal species isolated from pineapple-infected fruitlets and assayed in Koch’s postulates. (1) Colonies were incubated on potato dextrose agar (PDA) for 7 days at 27 °C in the dark. Each colony is shown from top (left) to bottom (right). (2) Conidia. *Fusarium sacchari* strains (**a**) BP138 and (**b**) BP575; *Fusarium ananatum* strain (**c**) BP383; *Fusarium oxysporum* strains (**d**) BP369 and (**e**) BP460; *Fusarium proliferatum* strains (**f**) BP114, (**g**) BP429, and (**h**) BP436; *Talaromyces stollii* strains (**i**) BP054, (**j**) BP185, and (**k**) BP462; *Talaromyces amestolkiae* strains (**l**) BP002, (**m**) BP257, and (**n**) BP605. Scale bar: 20 µm.

After 7 days of incubation on PDA Petri dishes, all species presented fast growth rates with a mean diameter (av. ± sd) reaching 7.25 cm, 7.77 ± 0.69 cm, 7.75 ± 0.70 cm, and 7.90 ± 0.19 cm for *F. ananatum*, *F. oxysporum*, *F. proliferatum*, and *F. sacchari*, respectively (Figure 10a1–h1). *T. stollii* and *T. amestolkiae* showed a slightly slower growth rate with respective mean diameters of 6.66 ± 0.08 cm and 6.68 ± 0.90 cm (Figure 10i1–n1). *Fusarium* colonies were circular with a thick and aerial white mycelium, except for strain BP575 (*F. sacchari*), which showed a short aerial mycelium. Species can be distinguished by their coloration when observed from a bottom range from orange (*F. sacchari*), to violet (*F. oxysporum*), to ochre/brown (*F. proliferatum*).

*Talaromyces* species showed circular to oval colonies with short and rough mycelium characterized by a green coloration in the oldest zone and with a white ring on the edges. The isolates identified as *T. amestolkiae* could be distinguished by a red pigmentation visible at the bottom of the Petri dishes. This was in addition to the secretion of a red exudate in the center of the colonies (Figure 10l1–n1). Microscopic observations of *Fusarium* species isolates showed straight (Figure 10a2–c2) and curved fusiform macroconidia (Figure 10d2,e2), while microconidia were ovoidal (Figure 10d2,e2) or ellipsoidal (Figure 10a2–c2,f2–h2). *Talaromyces amestolkiae* and *Talaromyces stollii* exhibited aggregated conidia with an ellipsoidal shape (Figure 10i2–n2).

**Figure 11:**
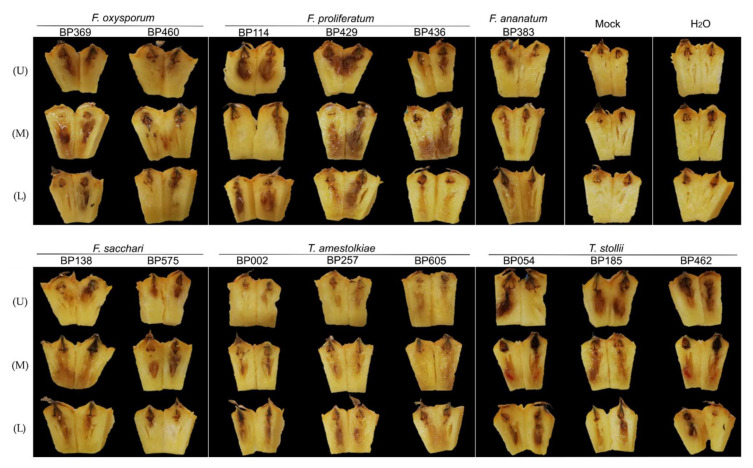
FCR symptoms observed 7 days post-inoculation (dpi) following controlled inoculations of five putative pathogenic fungal species in the blossom cup of fruitlets from the low (L), median (M), and upper (U) parts of ‘Queen Victoria’ pineapple fruit. *F. ananatum* strain BP383 was used as positive control; H_2_O: Sterile water-inoculated; mock: No inoculation.

Subsequently to sporulation on PDA, controlled inoculations were performed by injecting a normalized conidia solution into a blossom cup of fruitlets. After 7 days of incubation, fruitlets from low, median, and upper sections of pineapple fruit were extracted and cut into two equal parts. Samples inoculated with sterile water as well as the non-inoculated fruitlets (Mock) did not show any visible FCR symptom. All 5 tested species (13 isolates) led to the development of a vitreous aspect or black spot (Figure 11). Following *F. oxysporum* inoculations, black spot development was observed in both pineapple fruit sections. 

Similar observations were recorded for *F. sacchari*. Interestingly, *F. proliferatum* and *T. stollii* caused strong dark spot development in the fruitlet flesh. Through *T. stollii*-inoculated fruitlets, symptoms came out with a red pigmentation that was visible in samples from the median section only. Inoculations with 3 different isolates of *T. amestolkiae* led to slight necrosis associated with a vitreous appearance. Comparable symptoms were also reported after *F. ananatum* conidia injection, considered in this experiment as a positive control [10].

**Figure 12:**
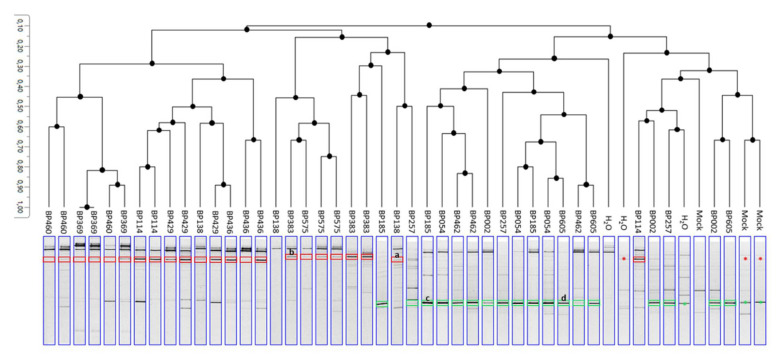
Dendrogram of PCR-DGGE profiles constructed by the UPGMA method. Red rectangles show *Fusarium* markers in *Fusarium*-inoculated fruitlets. Green rectangles show *Talaromyces* markers of *Talaromyces*-inoculated fruitlets. Red arrows indicate naturally present *Fusarium* sp. in control fruitlets. Green arrows indicate naturally present *Talaromyces* sp. in control fruitlets. BP138 and BP575: *Fusarium sacchari*; BP383: *Fusarium ananatum*; BP369 and BP460: *Fusarium oxysporum*; BP114, BP429, and BP436: *Fusarium proliferatum*; BP054, BP185, and BP462: *Talaromyces stollii*; BP002, BP257, and BP605: *Talaromyces amestolkiae*. H_2_O: sterile water-inoculated samples; mock: No inoculation. Letters label DNA bands, which were sequenced.

To fulfill Koch’s postulates, the potential causal agents were recovered by a molecular approach (PCR-DGGE) that allowed for a culture-independent characterization of microbial communities associated with a semi-quantitative determination of band patterns. To this end, *Fusarium*- and *Talaromyces*-inoculated fruitlets were distinguished, but the identification of species could not be achieved. First, a reference gel was performed on pure fungal strains using migration conditions that were similar to that of the inoculated samples (Figure S3). According to the UPGMA construction method, fungal patterns were structured depending on the genus of the tested strain (Figure 12). Two groups were then identified. One group contained *Fusarium*-inoculated samples and presented the ‘*Fusarium* marker’ according to the reference profiles. The second group was supported by *Talaromyces*-inoculated fruitlets with matching markers and control samples.

The DNA bands recovered from the gel were sequenced, and the identification results confirmed that the targeted bands corresponded to the *Fusarium* sp. and *Talaromyces* sp. that were used for inoculations (Table S3). Another difficulty relied on the presence of an important natural core mycobiome partly involving *Fusarium* and *Talaromyces* species. The fungal diversity of fruitlets could be appreciated in negative control samples that naturally presented fungal banding patterns corresponding to *Fusarium* (red asterisks) and *Talaromyces* (green asterisks) migration profiles. Fungi-inoculated samples, however, showed an intense band that was not detectable in symptom-free fruitlets that corresponded to the inoculated fungi.

## 4. Discussion

By analyzing pineapple cultivatable fungal flora, the composition of the mycobiome associated with FCR could be characterized. The data generated in the present study showed that an interaction between biotic and abiotic factors may impact disease spreading over a production area. Moreover, the determination of species contribution to FCR disease led to the discovery of new causal agents.

Our results demonstrate that structuration of the fungal flora of fruitlets was mainly composed of two species complexes that were recently characterized as *Fusarium fujikuroi* and *Talaromyces purpureogenus* [49,50]. This type of association was previously described in FCR through the isolation of *F. verticillioides* and *T. funiculosus* recovered from FCR symptoms [8,51], even though *T. funiculosus* had never been defined as prevalent in Reunion Island before. Previous sampling over eight production areas in the island showed a predominance of *F. ananatum* (72%), *F. oxysporum* (6.67%), *F. proliferatum* (0.67%), and *T. stollii* (20.67%) [52]. Conjointly, conidia with a fusiform shape were observed in a blossom cup of symptomless pineapples related to the ‘Queen Victoria’ cultivar, while ellipsoidal conidia were observed in the ‘MD-2’ defined as the FCR-resistant cultivar of reference [53]. However, the identification of species such as *Disporotrichum dimorphosporum*, *Galactomyces candidus*, and *Clavispora lusitaniae* in freshly cut pineapple (cv. ‘Queen Victoria’) revealed a higher level of fungal diversity in the fruit flesh [54].

By focusing on a specific plot environment, 49 fungal species were identified through 344 isolates. The infection level was reported over the three plot sections defined for the two experimental plots. Results showed that rows proximal to the mango orchard presented an elevated number of FCR symptoms, and this number was significantly lower in the rows proximal to the jackfruit trees. This suggested the existence of a disease gradient ranging from the mango orchard (high FCR incidence zone) to the jackfruit trees (low FCR incidence zone). Several soil-borne *Fusarium* species can cross long distances through water, especially irrigation water, under the form of chlamydospores. As an example, *F. oxysporum* causing *Fusarium* wilt of banana spread through the soil conductance, leading to pathogen invasion [55]. Nevertheless, the composition of the fungal flora of soil samples from both junctions was not correlated with the disease gradient observed in fruits over row sections. In fact, FCR pathogens such as *Fusarium* species were surprisingly mainly identified over the jackfruit tree interface, and members of the *Talaromyces* genus were poorly represented across soil samples. Mango orchard junctions only demonstrated the presence of *Talaromyces purpureogenus* (one of the four components of the *T. purpureogenus* species complex) as a potential new contributor to FCR. Interestingly, *F. proliferatum* and *F. sacchari* were previously identified as pathogens causing mango malformation disease and leading to an anarchic development of inflorescence [56,57]. Even though this pathology had never been reported in the various mango cultivars of this orchard, it is proposed that mango trees could represent a prevalent habitat for some FCR pathogens. In fact, across a landscape, fungi can switch from an endophyte lifestyle on a specific host to a pathogenic behavior in another host [58,59]. By considering the interaction between crop proximity (adjacent cultures were only 2 m away) and meteorological data, it was suggested that wind could help the dispersion of conidia from a mango tree canopy to pineapple rows, and it was hypothesized that FCR was an airborne disease. This profile also emphasized the significance of row orientation in relation to the main wind direction. The FCR gradient was slightly more pronounced in Plot 2, and the mean level of infestation per fruit was significantly higher. This suggested that the inoculum widespread from the mango tree canopy to the pineapple plot was carried by the wind further than for the plot having rows perpendicular to the major wind direction. In fact, the N-W/S-E-directed rows (Plot 1) showed a strong FCR gradient with a significant difference between plot sections. Thus, plants proximal to the mango orchard appeared as a ‘buffer zone’ for the shared pathogens and limited the wind dispersion of conidia over the plot. These observations are consistent with Parnell et al., who demonstrated that the spatial configuration of host in a landscape is the keystone determinant of plant-disease epidemics [60].

Among FCR pathogens, *F. ananatum* has been described the most. *F. ananatum* isolates from pineapples of Costa Rica and Ecuador showed a capacity to produce fumonisins B1 (FB_1_), fumonisins B2 (FB_2_), fumonisins B3 (FB_3_), and beauvericin (BEA). However, the production levels observed under in vitro conditions were only in low concentrations, thus constituting a limited risk for food contamination [61]. Following FCR sampling in Reunion Island, *F. proliferatum* and *F. oxysporum* have been described as mycotoxin producers of FB_1_, FB_2_, and BEA, while no toxigenic profile was detected for *T. stollii*. Significant amounts of FB_1_ and BEA have also been detected in naturally infected fruitlets in comparison to healthy ones [52]. It was thus suggested that these species could also be involved in FCR pathogenesis with a significant impact on food safety.

The characterization of the mycobiome related to both healthy and infected fruitlets resulted in the determination of significant pairwise co-occurrence between *Fusarium* and *Talaromyces* species. Although *F. ananatum* was described as the first causal agent of FCR in Reunion Island [11,52], our data showed a prevalence of *F. proliferatum* (50 strains), which seemed to define a hub over fungal interactions in pineapple. The prevalence of this species was also demonstrated following the sampling of diseased leaves and pineapple fruits in Malaysia [62]. Interestingly, this study identified *F. proliferatum* as the main causal agent of leaf spot disease and pineapple fruit rot, characterized by the development of brown necrosis in the fruit flesh and skin.

Across related abundance profiles and distribution patterns, *F. proliferatum*, *F. sacchari*, *F. oxysporum*, *T. stollii*, and *T. amestolkiae* were investigated for their pathogenicity. Following the injection of conidia in a blossom cup, all of these species showed a capacity to induce symptoms related to FCR. Several symptoms observed after seven days of incubation were only slightly pronounced, particularly for *F. oxysporum* and *T. amestolkiae*. This could be explained by the time variation for fungal development and symptom appearance, which may fluctuate according to the studied species. Surprisingly, the evolution of fungal flora of minimally processed pineapples also revealed the implication of *T. amestolkiae* in fruit spoilage over cold storage conditions [54]. Taken together, these elements illustrate that specific structural arrangement of microbial communities may lead to pathogenesis and/or to fruit quality depreciation [63]. We, therefore, propose the FCR pathosystem to be considered as multi-partite and hypothesize that other species related to *Fusarium fujikuroi* and *Talaromyces purpureogenus* species complexes could be involved. Thus, the presence of multiple pathogens in a production area may increase the epidemiological risks. This partially explained the difficulties faced over the past decades to elucidate the infectious process leading to pineapple FCR susceptibility.

Conventional verification of Koch’s postulates had to be adapted for pineapple fruits due to the constraints of dealing with non-microbial-free tissues for in vivo inoculation. As exposed with DGGE migration profiles, the initial (natural) microbial loads in fruitlets contain a high level of fungal diversity as numerous DNA bands that could be detected in symptomless fruitlets. As a comparison, 5–9 bands could be visualized in DGGE lanes of negative controls (H_2_O and Mock), while 1–5 species per fruitlets were isolated by a classical microbial procedure. The recovery of the pathogens from inoculated fruitlets was achieved following the establishment of reference migration profiles for each tested species. Over the 13 strains, strong DNA signals corresponded to a highly abundant inoculated species. This showed that the tested strains had been able to extend in the blossom cup of all biological replicates, except for one fruit inoculated with the *F. sacchari* (strain BP138). The inoculation of the tested species also modified the basal fungal communities. Numerous bands with various intensity were detected and contributed to the discrimination between *Fusarium*- and *Talaromyces*-inoculated mycobiomes. Interestingly, the fungal flora profiles of negative control samples were related to *Talaromyces*-inoculated samples rather than to *Fusarium*-inoculated fruitlets. This suggested a pathogen-specific evolution of the fruitlet mycobiome that is much more noticeable following inoculations with *Fusarium* species. The over-representation of these species may thus involve potentially synergistic or antagonistic microbial interactions. As an example, *F. verticillioides*, as a part of the endophytic fungal flora of maize, was described for its capacity to lower corn smut disease severity by limiting the biomass development of the pathogen *Ustilago maydis* [64]. In pineapple, the determination of positive pairwise co-occurrences led to the assumption that mycobiomes of healthy and naturally infected fruitlets were significantly correlated and that species pair combinations thus did not diverge from fruitlet phenotypes. Moreover, phylogenetic trees evidenced strong relationships among strains recovered from both HF and IF, proving that pathogenesis did not correlate with a fungal genetic polymorphism. Nevertheless, the interactions inside fungal communities are complex and may imply numerous species with various communication strategies, as observed in numerous studies [65,66,67,68]. It is important to consider that the present approach for the detected fungal species did not enable a determination of either the form (micro/macroconidia and mycelium) or their respective concentration in the fruitlets. The colonization of host tissues could be achieved by prevalent species forming an extensive mycelial network and could potentially modulate the development of specialized structures of the remaining fungal species [69]. The establishment of cooperative or competitive fungal interactions partly relies on chemical recognition [66,68]. Interestingly, *T. stollii* and *T. amestolkiae* were defined as red-pigment-producing fungi that correspond to azaphilone extrolites [70]. This corroborates the observations performed following the in vitro culture of *T. amestolkiae* and the in vivo development of *T. stollii* [50]. These secondary metabolites are related to antiviral, antimicrobial, antifungal, and other biological activities [71]. This illustrates the necessity to determine how chemical communication between fungi may contribute to FCR establishment. In addition, the present study focused on fungal flora without considering the role of yeast and bacteria, which may also influence the microbial dialog during pathogenesis. In tomato, Wei et al. showed that endophytic microbial communities sharing an ecological niche with the *Ralstonia solanacearum* pathogen exhibited a low disease incidence [72]. As frequently exemplified, *F. oxysporum* has been described for its capacities to secrete and accumulate fusaric acid in order to inhibit the production by *Pseudomonas fluorescens* of 2,4-diacetylphloroglucinol acting as an antibiotic [73]. Biological association between microbial species represented a source for the determination of biocontrol agents. In pineapple, the antagonistic activity of *Trichoderma asperellum* against *F. guttiforme* was already described for the control of fusariosis [74].

In addition, both relative abundance and spatial distribution of fungal species are also fluctuating in accordance with abiotic factors as precipitation, relative humidity, temperature, and wind speed [75]. In the present study, pineapples were cultivated during austral winter, which is considered the prevalent season for FCR incidence in Reunion Island [18]. The SIMPIÑA model was developed to predict the quality of ‘Queen Victoria’ following total soluble sugar estimation, agricultural practices, and production area [76]. Our data highlighted that the understanding of plant disease outbreaks requires the evaluation of both biotic and abiotic environments. *Fusarium* species belonging to the FFSC, such as *F. sacchari*, *F. verticillioides*, and *F. proliferatum*, were reported as causal agents of several tropical crops, including sugarcane and mango (as previously demonstrated), which constitute preponderant cultures in Reunion Island [77,78,79]. This supports the potential cross-contribution between shared pathogens and abiotic factors on fungal epidemics in tropical and subtropical regions. The evaluation of influence parameters and their interactions may lead to the definition of risk factors for FCR occurrence. These variables could then be incremented into the VICTORIA database to adapt disease management strategies to various plot contexts [80,81].

## 5. Conclusions

Our study provided a new understanding of the FCR pathosystem by demonstrating the implications of *F. proliferatum*, *F. oxysporum*, *F. sacchari*, *T. stollii*, and *T. amestolkiae* on pathogenesis. We demonstrated that the mycobiome composition of fruitlets is influenced by adjacent crops that share common pathogens and may cause elevated levels of FCR incidence. It appears essential to consider the plot environment, especially row orientation, in relation to the major wind direction. Finally, similar fungal populations were described in healthy and infected fruitlets, suggesting that pineapple susceptibility may be inherent to chemical communication between pathogens that modulate strategies of host tissue colonization. Determining in vitro and in vivo pathogens interacting-behaviors would be necessary to understand the virulence factors of FCR-pathogens and subsequently investigate the molecular crosstalk of host-multi-pathogens interaction.

## Figures and Tables

**Table 1:** Meteorological factors recorded from FIT (Flower Induction Treatment) in April 2018 to harvest in November 2018. *Source of satellite image: https://www.geoportail.gouv.fr/* (accessed on 28 January 2021).

	Min Temp (°C)	Average Temp (°C)	Max Temp (°C)	Average Rainfall (mm)	Number of Day(s) with Rain	Wind Direction	Max Gust Speed (km h^−1^)
April	20.12	23.19	27.85	15.78 *	10	south	116 *
May	18.42	22.20	27.48	0.66	5	east-south-east	85
June	17.10	20.68	25.72	2.47	8	south-east	73
July	15.53	19.50	24.64	4.46	12	south-east	80
August	17.07	21.10	26.60	0.00	0	south-east	75
September	16.64	21.15	27.60	0.15	1	south-east	80
October	17.65	21.75	27.79	3.85	8	east-south-east	88
November	18.98	23.49	28.66	0.03	1	south-east	69

(*) The tropical cyclone Fakir passed near the eastern coast of Reunion Island from 24 April to 26 April 2018.

**Table 3:** Identification of fungal species and their respective occurrence in healthy and naturally infected fruitlets.

Genus	Species	Occurrence		Top Match GenBank Accession Number * (*Frequency in Data*)
		Healthy Fruitlet (*n* = 96)	Infected Fruitlet (*n* = 96)	
*Fusarium*	*proliferatum*	28	22	KF993985.1 (*12*)
*Fusarium*	*ananatum*	19	18	MT010996.1 (*36*)
*Talaromyces*	*stollii*	8	16	JX315634.1 (*24*)
*Fusarium*	*verticillioides*	12	12	MT594370.1 (*10*)
*Talaromyces*	*amestolkiae*	13	9	KJ413360.1 (*13*)
*Fusarium*	*equiseti*	10	11	MN589630.1 (*4*)
*Phialemoniopsis*	*curvata*	6	12	AB278180.1 (*15*)
*Fusarium*	*oxysporum*	9	9	CP053267.1 (*4*)
*Fusarium*	*sp.*	10	5	JF740861.1 (*5*)
*Fusarium*	*solani*	5	8	MT594367.1 (*3*)/MK968891.1 (*3*)
*Talaromyces*	*purpureogenus*	5	5	KJ528885.1 (*3*)/MF476006.1 (*3*)
*Aspergillus*	*flavus*	2	6	MN955851.1 (*3*)
*Fusarium*	*incarnatum*	3	5	MK752398.1 (*2*)/MN882829.1 (*2*)
*Fusarium*	*fujikuroi*	6	1	MF281281.2 (*4*)
*Aspergillus*	*niger*	3	4	KY357318.1 (*2*)/MN788116.1 (*2*)
*Fusarium*	*chlamydosporum*	3	1	KJ125830.1 (*3*)
*Talaromyces*	*funiculosus*	2	2	AB893941.1 (*3*)
*Penicillium*	*sp.*	3	1	EU330619.1 (*2*)
*Pestalotiopsis*	*trachicarpicola*	2	2	MN295594.1 (*4*)
*Fusarium*	*sacchari*	0	3	MN193868.1 (*2*)
*Trichoderma*	*erinaceum*	0	2	MK109820.1 (*2*)
*Fusarium*	*napiforme*	1	1	MH862670.1 (*2*)
*Trichoderma*	*paraviridescens*	1	1	MF782827.1 (*1*)/MK418756.1 (*1*)
*Epicoccum*	*sorghinum*	1	1	MF782827.1 (*1*)/MK418756.1 (*1*)
*Trichoderma*	*sp.*	0	2	KX449479.1 (*1*)/MK870964.1 (*1*)
*Aspergillus*	*awamori*	2	0	KY416558.1 (*2*)
*Fusarium*	*falciforme*	2	0	MT251175.1 (*2*)
*Lasiodiplodia*	*mahajangana*	2	0	MH057188.1 (*2*)
*Trichoderma*	*asperellum*	0	1	KX538815.1 (*1*)
*Fusarium*	*circinatum*	0	1	MK334369.1 (*1*)
*Fusarium*	*cortaderiae*	0	1	AH012626.2 (*1*)
*Aspergillus*	*fumigatus*	0	1	MH844690.1 (*1*)
*Fusarium*	*graminearum*	0	1	MK460853.1 (*1*)
*Trichoderma*	*harzianum*	0	1	JN116710.1 (*1*)
*Diaporthe*	*kongii*	0	1	KR024740.1 (*1*)
*Curvularia*	*lunata*	0	1	MN971669.1 (*1*)
*Diaporthe*	*masirevicii*	0	1	MF668289.1 (*1*)
*Aspergillus*	*novoparasiticus*	0	1	MH279415.1 (*1*)
*Aspergillus*	*oryzae*	0	1	MN648727.1 (*1*)
*Rhizopus*	*stolonifer*	0	1	MF461025.1 (*1*)
*Trichoderma*	*trixiae*	0	1	MN889512.1 (*1*)
*Pestalotiopsis*	*vismiae*	0	1	KP747694.1 (*1*)
*Clonostachys*	*wenpingii*	0	1	NR_119651.1 (*1*)
*Cosmospora*	*butyri*	1	0	KU204560.1 (*1*)
*Lasiodiplodia*	*citricola*	1	0	KU530119.1 (*1*)
*Glomus*	*clarum*	1	0	AY035654.1 (*1*)
*Fusarium*	*dlamini*	1	0	MN173109.1 (*1*)
*Fusarium*	*ficicrescens*	1	0	KP662895.1 (*1*)
*Bionectria*	*ochroleuca*	1	0	EU552110.1 (*1*)
*Clonostachys*	*rosea*	1	0	MH047188.1 (*1*)
*Clonostachys*	*sp.*	1	0	MH681594.1 (*1*)
*Davidiella*	*sp.*	1	0	KX621979.1 (*1*)
*Diaporthe*	*sp.*	1	0	MH220834.1 (*1*)
*Lasiodiplodia*	*theobromae*	1	0	KR260829.1 (*1*)
*Aspergillus*	*welwitschiae*	1	0	MH374611.1 (*1*)

(*) The listed accession numbers correspond to the most frequent top match for each species.

## Supplementary Materials

The following are available online at https://www.mdpi.com/2309-608X/7/3/175/s1, Figure S1: Phylogenetic tree of *Fusarium* species computed by Maximum Likelihood (ML) analysis (JC+I+G as the best fit model) and based on the ITS sequences dataset. Only ML bootstrap branches that support values greater than 70% are shown. Strains isolated from infected and healthy fruitlets are indicated in red and black, respectively. The tree is rooted with *F. sporotrichoides* accession MT218410.1, Figure S2: Phylogenetic tree of *Talaromyces* species computed by Maximum Likelihood (ML) analysis (K80+G as the best fit model) and based on the ITS sequences dataset. Only ML bootstrap branches that support values greater than 70% are shown. Strains isolated from infected and healthy fruitlets are indicated in red and black, respectively. The tree is rooted with *T. cecidicola* accession MT182957.1, Figure S3: Reference DGGE profiles over ITS-1 region of DNA extracted from pure strains evaluated in Koch’s postulates. (Mix) indicates the combined migration of *F. ananatum* strain BP383, *F. sacchari* strain BP575, *F. oxysporum* strain BP460, *F. proliferatum* strain BP429, *T. stollii* strain BP054, and *T. amestolkiae* strain BP002, Table S1: Identification of fungal species isolated from healthy and naturally infected pineapple fruitlets, Table S2: Identification of fungal species isolated from soil coring points of cultures proximal to pineapple plots. Table S3: Identification of fungal species from DNA extracted from DGGE gels corresponding to *Fusarium*- and *Talaromyces*-inoculated pineapple fruitlets.
