# Supplementary material for: Pineapple Mycobiome Related to Fruitlet Core Rot Occurrence and the Influence of Fungal Species Dispersion Patterns
Source: J Fungi (Basel). 2021 Feb 28;7(3):175. doi: 10.3390/jof7030175 (PMC7997448; doi:10.3390/jof7030175)
Supplement: Supplementary file 1 [file jof-07-00175-s001.pdf]

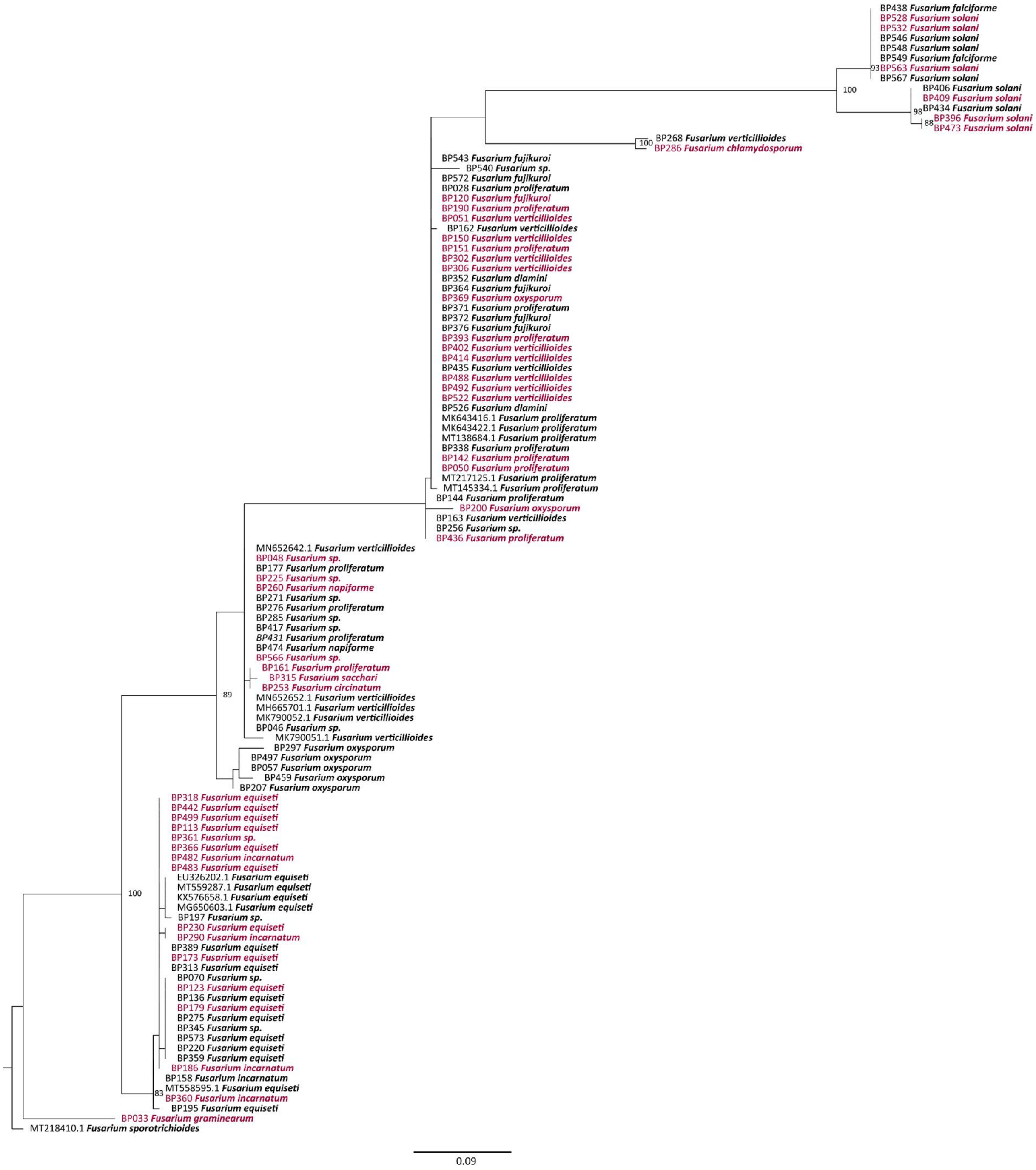

Figure S1. Phylogenetic tree of *Fusarium* species computed by Maximum Likelihood (ML) analysis (JC+I+G as the best fit model) and based on the ITS sequences dataset. Only ML bootstrap branches that support values greater than 70% are shown. Strains isolated from infected and healthy fruitlets are indicated in red and black respectively. The tree is rooted with *F. sporotrichoides* accession MT218410.1.

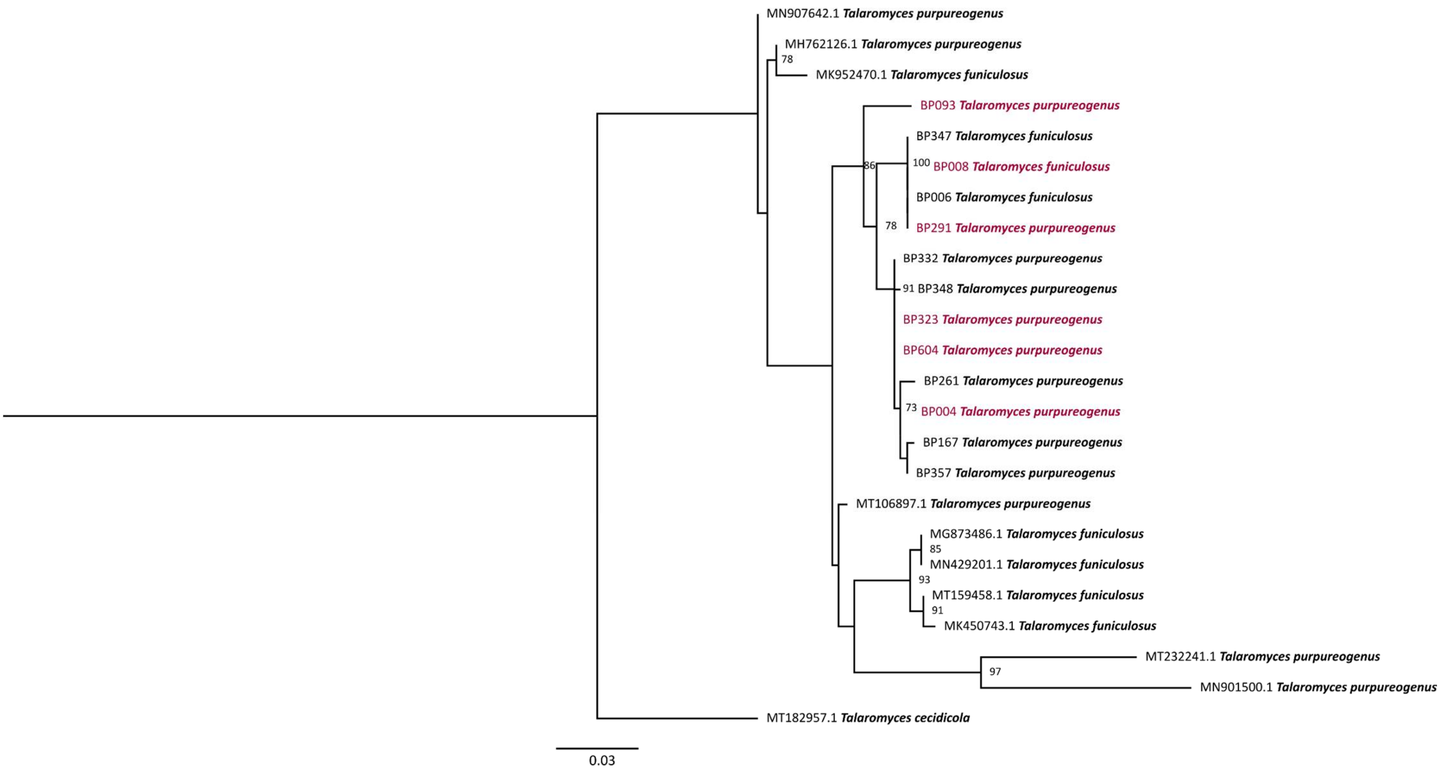

Figure S2. Phylogenetic tree of *Talaromyces* species computed by Maximum Likelihood (ML) analysis (K80+G as the best fit model) and based on the ITS sequences dataset. Only ML bootstrap branches that support values greater than 70% are shown. Strains isolated from infected and healthy fruitlets are indicated in red and black respectively. The tree is rooted with *T. cecidicola* accession MT182957.1.

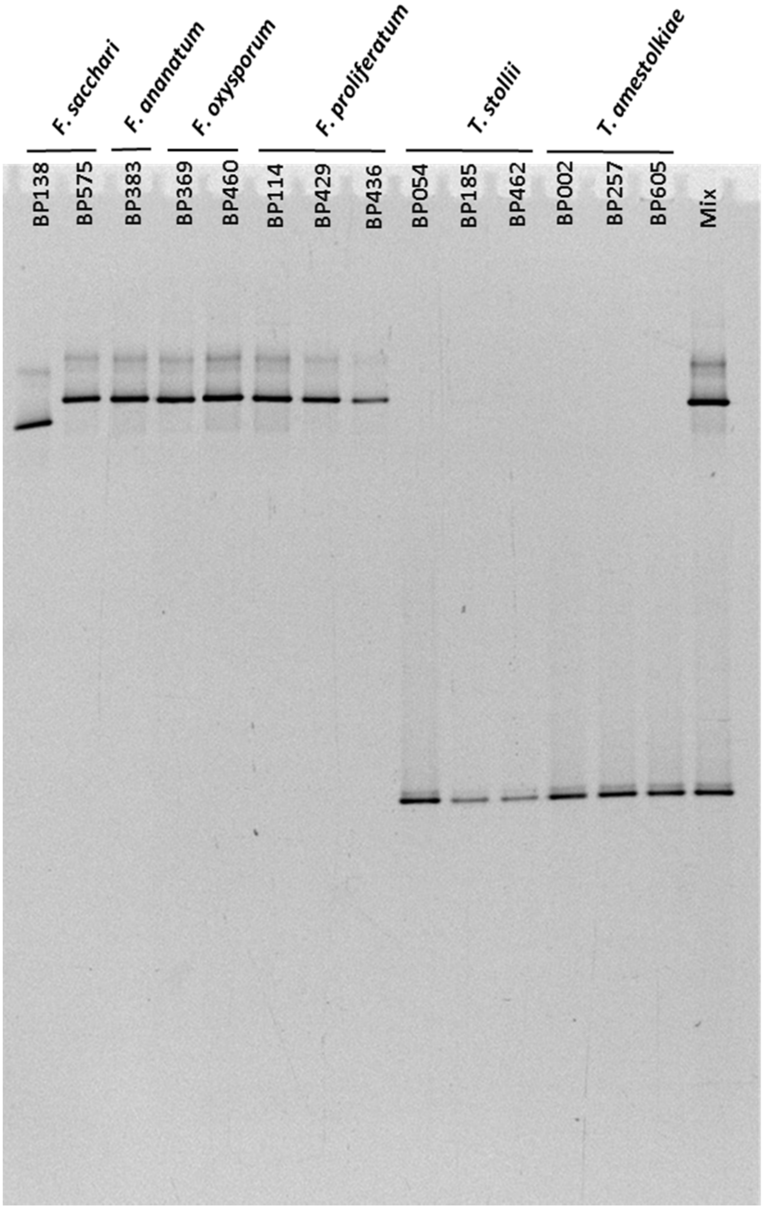

Figure S3. Reference DGGE profiles over ITS-1 region of DNA extracted from pure strains evaluated in Koch's postulates. (Mix) indicates the combined migration of *F. ananatum* strain BP383, *F. sacchari* strain BP575, *F. oxysporum* strain BP460, *F. proliferatum* strain BP429, *T. stollii* strain BP054, and *T. amestolkiae* strain BP002.

Table S1. Identification of fungal species isolated from healthy and naturally infected pineapple fruitlets.

Isolate ID	Identification	Sampled tissue	Fragment size (bp)	Identity (%)	E-value	Locus	GenBank accession number
BP001	*Talaromyces stollii*	healthy fruitlet	398	100	0	β-tubulin	JX315634.1
BP002	*Talaromyces amestolkiae*	infected fruitlet	400	99.25	0	β-tubulin	MH287207.1
BP003	*Talaromyces amestolkiae*	infected fruitlet	414	100	0	β-tubulin	KJ413360.1
BP004	*Talaromyces purpureogenus*	infected fruitlet	567	99.47	0	ITS	MF475916.1
BP006	*Talaromyces funiculosus*	healthy fruitlet	562	99.64	0	ITS	AB893941.1
BP008	*Talaromyces funiculosus*	infected fruitlet	548	99.64	0	ITS	AB893941.1
BP012	*Talaromyces stollii*	infected fruitlet	320	100	4,00E-165	β-tubulin	JX965359.1
BP014	*Talaromyces stollii*	infected fruitlet	406	100	0	β-tubulin	JX315634.1
BP016	*Talaromyces stollii*	healthy fruitlet	406	100	0	β-tubulin	JX315634.1
BP018	*Talaromyces stollii*	healthy fruitlet	406	100	0	β-tubulin	JX315634.1
BP019	*Talaromyces stollii*	infected fruitlet	406	100	0	β-tubulin	JX315634.1
BP020	*Talaromyces stollii*	infected fruitlet	406	100	0	β-tubulin	JX315634.1
BP022	*Talaromyces stollii*	infected fruitlet	406	100	0	β-tubulin	JX315634.1
BP025	*Talaromyces amestolkiae*	healthy fruitlet	414	100	0	β-tubulin	MH287207.1
BP027	*Trichoderma trixiae*	infected fruitlet	134	100	5,00E-62	ITS	MN889512.1
BP028	*Fusarium proliferatum*	healthy fruitlet	532	99,81	0	ITS	MN170571.1
BP029	*Trichoderma paraviridescens*	infected fruitlet	587	100	0	ITS	MK418756.1
BP030	*Aspergillus niger*	infected fruitlet	579	100	0	ITS	JF439461.1
BP031	*Pestalotiopsis vismiae*	infected fruitlet	587	99,66	0	ITS	KP747694.1
BP033	*Fusarium graminearum*	infected fruitlet	514	100	0	ITS	MK460853.1
BP034	*Talaromyces stollii*	healthy fruitlet	405	100	0	β-tubulin	JX315634.1
BP035	*Fusarium cortaderiae*	infected fruitlet	290	99,66	4,00E-148	β-tubulin	AH012626.2
BP037	*Trichoderma sp.*	infected fruitlet	596	99.83	0	ITS	KX449479.1
BP038	*Rhizopus stolonifer*	infected fruitlet	788	100	0	ITS	MF461025.1
BP039	*Fusarium chlamydosporum*	healthy fruitlet	272	100	1,00E-139	β-tubulin	KJ125830.1
BP041	*Epicoccum sorghinum*	infected fruitlet	298	99,33	2,00E-150	β-tubulin	MF987525.1
BP042	*Trichoderma harzianum*	infected fruitlet	565	100	0	ITS	JN116710.1
BP043	*Aspergillus niger*	healthy fruitlet	450	99,56	0	ITS	MN788116.1
BP044	*Fusarium proliferatum*	healthy fruitlet	665	100	0.0	EF1α	KF993985.1
BP046	*Fusarium sp.*	healthy fruitlet	528	100	0	ITS	HQ630965.1
BP048	*Fusarium sp.*	infected fruitlet	539	100	0	ITS	HQ630965.1
BP050	*Fusarium proliferatum*	infected fruitlet	544	99,26	0	ITS	MN170571.1
BP051	*Fusarium verticillioides*	infected fruitlet	531	100	0	ITS	KR183784.1
BP054	*Talaromyces stollii*	infected fruitlet	406	99,51	0	β-tubulin	JX315634.1
BP057	*Fusarium oxysporum*	healthy fruitlet	524	100	0	ITS	MH752745.1
BP059	*Aspergillus awamori*	healthy fruitlet	505	100	0	β-tubulin	KY416558.1
BP061	*Fusarium ananatum*	healthy fruitlet	632	100	0	EF1α	MT010996.1
BP062	*Talaromyces funiculosus*	infected fruitlet	414	99,52	0	β-tubulin	MK208457.1
BP063	*Epicoccum sorghinum*	healthy fruitlet	312	99,36	0	β-tubulin	MF987525.1
BP065	*Fusarium ananatum*	healthy fruitlet	663	100	0	EF1α	MT010996.1
BP070	*Fusarium sp.*	healthy fruitlet	523	99,81	0	ITS	MG664720.1
BP071	*Trichoderma paraviridescens*	healthy fruitlet	586	100	0	ITS	MF782827.1
BP072	*Fusarium proliferatum*	healthy fruitlet	668	100	0	EF1α	KU872092.1
BP076	*Fusarium proliferatum*	healthy fruitlet	669	99,85	0	EF1α	KF993985.1
BP077	*Talaromyces amestolkiae*	healthy fruitlet	422	100	0	β-tubulin	MH287207.1
BP078	*Pestalotiopsis trachicarpicola*	healthy fruitlet	440	100	0	β-tubulin	MN295594.1
BP081	*Talaromyces stollii*	healthy fruitlet	398	100	0	β-tubulin	JX315634.1
BP082	*Talaromyces amestolkiae*	infected fruitlet	401	100	0	β-tubulin	MH287207.1
BP084	*Aspergillus niger*	healthy fruitlet	508	100	0	β-tubulin	HQ632734.1

Table S1. Continued

Isolate ID	Identification	Sampled tissue	Fragment size (bp)	Identity (%)	E-value	Locus	GenBank accession number
BP086	*Talaromyces amestolkiae*	healthy fruitlet	414	100	0	β-tubulin	KJ413360.1
BP087	*Talaromyces stollii*	infected fruitlet	406	99,75	0	β-tubulin	JX315634.1
BP088	*Talaromyces stollii*	infected fruitlet	398	100	0	β-tubulin	JX315634.1
BP091	*Trichoderma asperellum*	infected fruitlet	43	100	1,00E-13	ITS	KX538815.1
BP093	*Talaromyces purpureogenus*	infected fruitlet	523	100	0	ITS	MH185977.1
BP096	*Pestalotiopsis trachicarpicola*	infected fruitlet	440	100	0	β-tubulin	MN295594.1
BP097	*Talaromyces amestolkiae*	infected fruitlet	422	100	0	β-tubulin	KJ413360.1
BP100	*Aspergillus niger*	infected fruitlet	573	100	0	ITS	KY357318.1
BP101	*Fusarium ananatum*	infected fruitlet	666	100	0	EF1α	MT010996.1
BP102	*Talaromyces amestolkiae*	infected fruitlet	408	100	0	β-tubulin	KJ413360.1
BP104	*Talaromyces amestolkiae*	infected fruitlet	408	100	0	β-tubulin	LT559068.1
BP106	*Clonostachys wenpingii*	infected fruitlet	496	100	0	ITS	NR_119651.1
BP107	*Aspergillus flavus*	infected fruitlet	424	100	0	ITS	MN955851.1
BP109	*Fusarium proliferatum*	healthy fruitlet	659	100	0	EF1α	KF993985.1
BP113	*Fusarium equiseti*	infected fruitlet	514	100	0	ITS	MK680159.1
BP114	*Fusarium proliferatum*	infected fruitlet	658	100	0	EF1α	KF993985.1
BP118	*Fusarium proliferatum*	healthy fruitlet	655	99,85	0	EF1α	MN386746.1
BP120	*Fusarium fujikuroi*	infected fruitlet	527	100	0	ITS	MF281281.2
BP121	*Fusarium ananatum*	infected fruitlet	646	100	0	EF1α	MT010996.1
BP123	*Fusarium equiseti*	infected fruitlet	511	100	0	ITS	KF624787.1
BP127	*Fusarium proliferatum*	infected fruitlet	646	100	0	EF1α	MN386746.1
BP129	*Trichoderma sp.*	infected fruitlet	598	99,83	0	ITS	MK870964.1
BP130	*Talaromyces amestolkiae*	healthy fruitlet	414	100	0	β-tubulin	KJ413360.1
BP136	*Fusarium equiseti*	healthy fruitlet	516	100	0	ITS	KF624787.1
BP138	*Fusarium sacchari*	infected fruitlet	663	100	0	EF1α	MN193868.1
BP139	*Clonostachys sp.*	healthy fruitlet	503	100	0	ITS	MH681594.1
BP141	*Bionectria ochroleuca*	healthy fruitlet	550	100	0	ITS	EU552110.1
BP144	*Fusarium proliferatum*	healthy fruitlet	528	100	0	ITS	MH712288.1
BP147	*Fusarium proliferatum*	infected fruitlet	644	99,84	0	EF1α	MN175178.1
BP148	*Fusarium proliferatum*	healthy fruitlet	654	99,85	0	EF1α	KU872092.1
BP150	*Fusarium verticillioides*	infected fruitlet	537	99,63	0	ITS	MT594370.1
BP151	*Fusarium proliferatum*	infected fruitlet	523	100	0	ITS	MK336501.1
BP154	*Fusarium incarnatum*	infected fruitlet	292	99,66	3,00E-149	β-tubulin	MK439850.1
BP155	*Fusarium proliferatum*	healthy fruitlet	644	99,84	0	EF1α	KF993985.1
BP156	*Fusarium proliferatum*	healthy fruitlet	663	100	0	EF1α	KF993985.1
BP158	*Fusarium incarnatum*	healthy fruitlet	525	99.81	0	ITS	MN882829.1
BP159	*Fusarium proliferatum*	healthy fruitlet	663	100	0	EF1α	FJ538242.1
BP161	*Fusarium proliferatum*	infected fruitlet	531	99,62	0	ITS	MH591759.1
BP162	*Fusarium verticillioides*	healthy fruitlet	541	99,82	0	ITS	MT594370.1
BP163	*Fusarium verticillioides*	healthy fruitlet	527	100	0	ITS	MT126609.1
BP165	*Fusarium proliferatum*	healthy fruitlet	292	99,66	1,00E-148	β-tubulin	MH398186.1
BP167	*Talaromyces purpureogenus*	healthy fruitlet	513	99,61	0	ITS	MF476006.1
BP169	*Glomus clarum*	healthy fruitlet	102	100	0	ITS	AY035654.1
BP170	*Diaporthe masirevicii*	infected fruitlet	487	99,79	0	β-tubulin	MF668289.1
BP173	*Fusarium equiseti*	infected fruitlet	512	99,8	0	ITS	MG650603.1
BP177	*Fusarium proliferatum*	healthy fruitlet	530	100	0	ITS	MH591759.1
BP179	*Fusarium equiseti*	infected fruitlet	523	100	0	ITS	KF624787.1
BP180	*Fusarium proliferatum*	infected fruitlet	657	100	0	EF1α	MK442093.1
BP185	*Talaromyces stollii*	infected fruitlet	398	100	0	β-tubulin	JX315634.1
BP186	*Fusarium incarnatum*	infected fruitlet	467	100	0	ITS	MK336548.1

Table S1. Continued

Isolate ID	Identification	Sampled tissue	Fragment size (bp)	Identity (%)	E-value	Locus	GenBank accession number
BP187	*Fusarium ananatum*	healthy fruitlet	306	98,39	1,00E-144	β-tubulin	LC386036.1
BP188	*Fusarium ananatum*	infected fruitlet	644	100	0	EF1α	MT010996.1
BP190	*Fusarium proliferatum*	infected fruitlet	539	99,81	0	ITS	MH055399.1
BP191	*Fusarium equiseti*	healthy fruitlet	294	100	5,00E-152	β-tubulin	MK278900.1
BP194	*Talaromyces stollii*	infected fruitlet	398	100	0	β-tubulin	JX315634.1
BP195	*Fusarium equiseti*	healthy fruitlet	512	99.80	0	ITS	MN589630.1
BP197	*Fusarium sp.*	healthy fruitlet	517	100	0	ITS	MG664720.1
BP199	*Aspergillus fumigatus*	infected fruitlet	504	100	0	β-tubulin	MH844690.1
BP200	*Fusarium oxysporum*	infected fruitlet	88	98.86	6,00E-36	ITS	MK729614.1
BP202	*Aspergillus niger*	healthy fruitlet	543	100	0	ITS	MK336494.1
BP204	*Fusarium oxysporum*	infected fruitlet	658	100	0	EF1α	CP053267.1
BP207	*Fusarium oxysporum*	infected fruitlet	527	100	0	ITS	KU847855.1
BP208	*Fusarium ficicrescens*	healthy fruitlet	286	99,3	7,00E-143	β-tubulin	KP662895.1
BP210	*Talaromyces amestolkiae*	healthy fruitlet	408	100	0	β-tubulin	LT559068.1
BP211	*Talaromyces stollii*	infected fruitlet	398	100	0	β-tubulin	JX315634.1
BP212	*Pestalotiopsis trachicarpicola*	infected fruitlet	434	100	0	β-tubulin	MN295594.1
BP213	*Talaromyces stollii*	infected fruitlet	398	100	0	β-tubulin	JX315634.1
BP215	*Talaromyces stollii*	healthy fruitlet	398	100	0	β-tubulin	JX315634.1
BP216	*Pestalotiopsis trachicarpicola*	healthy fruitlet	441	100	0	β-tubulin	MN295594.1
BP218	*Fusarium proliferatum*	healthy fruitlet	663	99,85	0	EF1α	KF993985.1
BP219	*Fusarium oxysporum*	healthy fruitlet	660	100	0	EF1α	MG000154.1
BP220	*Fusarium equiseti*	healthy fruitlet	511	100	0	ITS	MN589630.1
BP222	*Fusarium proliferatum*	infected fruitlet	661	100	0	EF1α	KF993985.1
BP224	*Fusarium chlamydosporum*	healthy fruitlet	272	100	2,00E-138	β-tubulin	KJ125830.1
BP225	*Fusarium sp.*	infected fruitlet	528	100	0	ITS	HQ630965.1
BP230	*Fusarium equiseti*	infected fruitlet	487	99,79	0	ITS	MN589630.1
BP236	*Fusarium ananatum*	infected fruitlet	664	100	0	EF1α	MT010996.1
BP238	*Fusarium ananatum*	infected fruitlet	641	100	0	EF1α	MT010996.1
BP240	*Talaromyces stollii*	infected fruitlet	392	100	0	β-tubulin	JX315634.1
BP241	*Fusarium ananatum*	healthy fruitlet	652	99,85	0	EF1α	MT010996.1
BP243	*Fusarium ananatum*	healthy fruitlet	656	100	0	EF1α	MT010996.1
BP247	*Fusarium ananatum*	healthy fruitlet	653	100	0	EF1α	MT010996.1
BP251	*Talaromyces amestolkiae*	healthy fruitlet	408	100	0	β-tubulin	MH287207.1
BP253	*Fusarium circinatum*	infected fruitlet	515	100	0	ITS	MK334369.1
BP254	*Aspergillus novoparasiticus*	infected fruitlet	562	100	0	ITS	MH279415.1
BP256	*Fusarium sp.*	healthy fruitlet	517	100	0	ITS	MK355727.1
BP257	*Talaromyces amestolkiae*	infected fruitlet	407	100	0	β-tubulin	KJ413360.1
BP259	*Penicillium sp.*	healthy fruitlet	563	100	0	ITS	EU330619.1
BP260	*Fusarium napiforme*	infected fruitlet	515	100	0	ITS	MH862670.1
BP261	*Talaromyces purpureogenus*	healthy fruitlet	502	99,8	0	ITS	MF476006.1
BP263	*Diaporthe sp.*	healthy fruitlet	461	98,05	0	β-tubulin	MH220834.1
BP265	*Talaromyces amestolkiae*	healthy fruitlet	401	100	0	β-tubulin	KJ413360.1
BP268	*Fusarium verticillioides*	healthy fruitlet	543	100	0	ITS	MH863977.1
BP269	*Aspergillus awamori*	healthy fruitlet	506	100	0	β-tubulin	KY416558.1
BP271	*Fusarium sp.*	healthy fruitlet	506	100	0	ITS	MH777054.1
BP273	*Talaromyces amestolkiae*	healthy fruitlet	421	100	0	β-tubulin	KJ413360.1
BP275	*Fusarium equiseti*	healthy fruitlet	511	100	0	ITS	MN589627.1
BP276	*Fusarium proliferatum*	healthy fruitlet	529	100	0	ITS	MH591759.1
BP278	*Fusarium oxysporum*	healthy fruitlet	645	100	0	EF1α	CP053267.1
BP280	*Fusarium incarnatum*	healthy fruitlet	251	100	8,00E-127	β-tubulin	MK439849.1

Table S1. Continued

Isolate ID	Identification	Sampled tissue	Fragment size (bp)	Identity (%)	E-value	Locus	GenBank accession number
BP285	*Fusarium sp.*	healthy fruitlet	514	100	0	ITS	MT530119.1
BP286	*Fusarium chlamydosporum*	infected fruitlet	533	99,81	0	ITS	MG250448.1
BP288	*Diaporthe kongii*	infected fruitlet	544	99,26	0	ITS	KR024740.1
BP290	*Fusarium incarnatum*	infected fruitlet	496	99,8	0	ITS	MN882829.1
BP291	*Talaromyces purpureogenus*	infected fruitlet	547	99,63	0	ITS	MK749843.1
BP293	*Fusarium proliferatum*	healthy fruitlet	676	100	0	EF1α	KF993985.1
BP296	*Fusarium equiseti*	healthy fruitlet	294	99,66	5,00E-149	β-tubulin	MK278900.1
BP297	*Fusarium oxysporum*	infected fruitlet	364	100	0	ITS	EU273520.1
BP302	*Fusarium verticillioides*	infected fruitlet	529	100	0	ITS	MT594370.1
BP305	*Fusarium ananatum*	healthy fruitlet	659	100	0	EF1α	MT010996.1
BP306	*Fusarium verticillioides*	infected fruitlet	541	99,82	0	ITS	MT594370.1
BP308	*Fusarium ananatum*	infected fruitlet	671	100	0	EF1α	MT010996.1
BP313	*Fusarium equiseti*	healthy fruitlet	506	99,8	0	ITS	MN589629.1
BP315	*Fusarium sacchari*	infected fruitlet	438	100	0	ITS	KC464631.1
BP316	*Lasiodiplodia mahajangana*	healthy fruitlet	398	100	0	ITS	MH057188.1
BP318	*Fusarium equiseti*	infected fruitlet	514	100	0	ITS	MH578585.1
BP319	*Fusarium proliferatum*	healthy fruitlet	658	100	0	EF1α	KU872092.1
BP320	*Talaromyces amestolkiae*	healthy fruitlet	414	100	0	β-tubulin	KJ413360.1
BP323	*Talaromyces purpureogenus*	infected fruitlet	566	100	0	ITS	MF475966.1
BP324	*Fusarium proliferatum*	healthy fruitlet	659	100	0	EF1α	MK442093.1
BP326	*Fusarium ananatum*	infected fruitlet	671	100	0	EF1α	MT010996.1
BP331	*Talaromyces amestolkiae*	healthy fruitlet	414	100	0	β-tubulin	KJ413360.1
BP332	*Talaromyces purpureogenus*	healthy fruitlet	556	99,82	0	ITS	KJ528885.1
BP334	*Talaromyces stollii*	infected fruitlet	398	100	0	β-tubulin	JX315634.1
BP335	*Curvularia lunata*	infected fruitlet	533	100	0	ITS	MN971669.1
BP338	*Fusarium proliferatum*	healthy fruitlet	538	100	0	ITS	MT466521.1
BP340	*Talaromyces stollii*	healthy fruitlet	398	100	0	β-tubulin	JX315634.1
BP341	*Penicillium sp.*	infected fruitlet	576	99,48	0	ITS	KF931337.1
BP342	*Fusarium ananatum*	healthy fruitlet	661	100	0	EF1α	MT010996.1
BP343	*Talaromyces amestolkiae*	healthy fruitlet	422	99,29	0	β-tubulin	KJ413360.1
BP344	*Talaromyces stollii*	infected fruitlet	398	100	0	β-tubulin	JX315634.1
BP345	*Fusarium sp.*	healthy fruitlet	511	100	0	ITS	KM979504.1
BP346	*Talaromyces stollii*	healthy fruitlet	396	99,75	0	β-tubulin	JX315634.1
BP347	*Talaromyces funiculosus*	healthy fruitlet	501	99,6	0	ITS	AB893941.1
BP348	*Talaromyces purpureogenus*	healthy fruitlet	556	99,82	0	ITS	KJ528885.1
BP349	*Penicillium sp.*	healthy fruitlet	557	99,82	0	ITS	EU330619.1
BP351	*Trichoderma erinaceum*	infected fruitlet	228	100	2,00E-115	ITS	MK109820.1
BP352	*Fusarium dlamini*	infected fruitlet	548	100	0	ITS	MN173109.1
BP353	*Fusarium ananatum*	infected fruitlet	656	100	0	EF1α	MT010996.1
BP356	*Fusarium ananatum*	healthy fruitlet	656	100	0	EF1α	MT010996.1
BP357	*Talaromyces purpureogenus*	healthy fruitlet	513	99.81	0	ITS	MF476006.1
BP359	*Fusarium equiseti*	healthy fruitlet	487	100	0	ITS	MN589627.1
BP360	*Fusarium incarnatum*	infected fruitlet	471	100	0	ITS	MN882828.1
BP361	*Fusarium sp.*	infected fruitlet	527	99,81	0	ITS	EU330623.1
BP364	*Fusarium fujikuroi*	healthy fruitlet	533	100	0	ITS	MF281281.2
BP366	*Fusarium equiseti*	infected fruitlet	514	100	0	ITS	MK680159.1
BP367	*Fusarium ananatum*	infected fruitlet	669	100	0	EF1α	MT010996.1
BP368	*Fusarium oxysporum*	infected fruitlet	663	99,7	0	EF1α	MN386739.1
BP369	*Fusarium oxysporum*	infected fruitlet	539	100	0	ITS	KC119203.1
BP370	*Aspergillus flavus*	infected fruitlet	336	100	0	ITS	MF152938.1

Table S1. Continued

Isolate ID	Identification	Sampled tissue	Fragment size (bp)	Identity (%)	E-value	Locus	GenBank accession number
BP371	*Fusarium proliferatum*	healthy fruitlet	538	100	0	ITS	MH055399.1
BP372	*Fusarium fujikuroi*	healthy fruitlet	539	100	0	ITS	MF281281.2
BP373	*Fusarium proliferatum*	infected fruitlet	671	100	0	EF1α	MK442093.1
BP376	*Fusarium fujikuroi*	healthy fruitlet	531	100	0	ITS	MF281281.2
BP379	*Fusarium ananatum*	healthy fruitlet	658	100	0	EF1α	MT010996.1
BP383	*Fusarium ananatum*	infected fruitlet	656	100	0	EF1α	MT010996.1
BP384	*Fusarium ananatum*	infected fruitlet	655	100	0	EF1α	MT010996.1
BP388	*Fusarium ananatum*	healthy fruitlet	671	100	0	EF1α	MT010996.1
BP389	*Fusarium equiseti*	infected fruitlet	507	100	0	ITS	MH879584.1
BP391	*Fusarium proliferatum*	infected fruitlet	660	100	0	EF1α	MN539103.1
BP393	*Fusarium proliferatum*	infected fruitlet	531	99,81	0	ITS	MH055399.1
BP395	*Fusarium solani*	infected fruitlet	686	100	0	EF1α	MN200289.1
BP396	*Fusarium solani*	infected fruitlet	549	100	0	ITS	MK968891.1
BP400	*Fusarium ananatum*	healthy fruitlet	669	100	0	EF1α	MT010996.1
BP402	*Fusarium verticillioides*	infected fruitlet	526	100	0	ITS	MT594370.1
BP405	*Talaromyces amestolkiae*	healthy fruitlet	414	100	0	β-tubulin	MH287203.1
BP406	*Fusarium solani*	healthy fruitlet	548	100	0	ITS	JX173101.1
BP407	*Fusarium ananatum*	infected fruitlet	670	100	0	EF1α	MT010996.1
BP409	*Fusarium solani*	infected fruitlet	526	100	0	ITS	KF030977.1
BP410	*Fusarium ananatum*	healthy fruitlet	673	100	0	EF1α	MT010996.1
BP413	*Fusarium proliferatum*	healthy fruitlet	669	99,85	0	EF1α	KF993985.1
BP414	*Fusarium verticillioides*	infected fruitlet	524	100	0	ITS	MT594370.1
BP416	*Talaromyces amestolkiae*	infected fruitlet	406	100	0	β-tubulin	KJ413360.1
BP417	*Fusarium sp.*	healthy fruitlet	513	100	0	ITS	MH777054.1
BP418	*Aspergillus niger*	infected fruitlet	569	100	0	ITS	KY357318.1
BP420	*Fusarium verticillioides*	healthy fruitlet	662	100	0	EF1α	MN861780.1
BP421	*Fusarium verticillioides*	infected fruitlet	661	100	0	EF1α	MT095057.1
BP422	*Fusarium ananatum*	healthy fruitlet	636	100	0	EF1α	MT010996.1
BP423	*Fusarium ananatum*	infected fruitlet	656	100	0	EF1α	MT010996.1
BP426	*Fusarium incarnatum*	infected fruitlet	290	99,66	4,00E-148	β-tubulin	MK752398.1
BP428	*Fusarium proliferatum*	infected fruitlet	661	100	0	EF1α	MN539103.1
BP429	*Fusarium proliferatum*	infected fruitlet	676	100	0	EF1α	FJ538242.1
BP430	*Fusarium ananatum*	infected fruitlet	659	100	0	EF1α	MT010996.1
BP431	*Fusarium proliferatum*	healthy fruitlet	512	100	0	ITS	MT563410.1
BP432	*Cosmospora butyri*	healthy fruitlet	530	100	0	ITS	KU204560.1
BP433	*Fusarium proliferatum*	healthy fruitlet	283	100	7,00E-146	β-tubulin	MH398186.1
BP434	*Fusarium solani*	healthy fruitlet	532	100	0	ITS	MK968891.1
BP435	*Fusarium verticillioides*	healthy fruitlet	541	100	0	ITS	MT594370.1
BP436	*Fusarium proliferatum*	infected fruitlet	530	100	0	ITS	MH712288.1
BP438	*Fusarium falciforme*	healthy fruitlet	547	100	0	ITS	MT251175.1
BP441	*Phialemoniopsis curvata*	infected fruitlet	531	99,26	0	ITS	AB278180.1
BP442	*Fusarium equiseti*	infected fruitlet	498	100	0	ITS	MH578585.1
BP443	*Fusarium ananatum*	infected fruitlet	659	100	0	EF1α	MT010996.1
BP444	*Fusarium sp.*	healthy fruitlet	651	98,79	0	EF1α	JF740861.1
BP445	*Fusarium oxysporum*	healthy fruitlet	676	100	0	EF1α	MN417196.1
BP447	*Fusarium ananatum*	healthy fruitlet	656	100	0	EF1α	MT010996.1
BP449	*Fusarium oxysporum*	healthy fruitlet	675	99,7	0	EF1α	MN417202.1
BP450	*Fusarium incarnatum*	healthy fruitlet	306	99.35	1,00E-154	β-tubulin	MK752398.1
BP454	*Fusarium oxysporum*	healthy fruitlet	666	100	0	EF1α	CP053267.1
BP458	*Fusarium oxysporum*	healthy fruitlet	671	100	0	EF1α	MN507111.1

Table S1. Continued

Isolate ID	Identification	Sampled tissue	Fragment size (bp)	Identity (%)	E-value	Locus	GenBank accession number
BP459	*Fusarium oxysporum*	infected fruitlet	454	99,34	0	ITS	MH777057.1
BP460	*Fusarium oxysporum*	infected fruitlet	666	100	0	EF1α	MN507111.1
BP462	*Talaromyces stollii*	infected fruitlet	398	100	0	β-tubulin	JX315634.1
BP463	*Fusarium ananatum*	infected fruitlet	664	100	0	EF1α	MT010996.1
BP465	*Fusarium ananatum*	infected fruitlet	661	100	0	EF1α	MT010996.1
BP468	*Fusarium ananatum*	healthy fruitlet	483	99,44	0	EF1α	MT010996.1
BP469	*Fusarium verticillioides*	healthy fruitlet	662	100	0	EF1α	MN861780.1
BP473	*Fusarium solani*	infected fruitlet	508	100	0	ITS	MK968891.1
BP474	*Fusarium napiforme*	healthy fruitlet	517	100	0	ITS	MH685904.1
BP475	*Fusarium verticillioides*	healthy fruitlet	659	100	0	EF1α	MT095057.1
BP476	*Fusarium oxysporum*	healthy fruitlet	290	100	9,00E-150	β-tubulin	MF806523.1
BP477	*Fusarium proliferatum*	infected fruitlet	660	100	0	EF1α	MN386746.1
BP478	*Fusarium proliferatum*	infected fruitlet	661	100	0	EF1α	MN386746.1
BP482	*Fusarium incarnatum*	infected fruitlet	526	100	0	ITS	MH712152.1
BP483	*Fusarium equiseti*	infected fruitlet	524	100	0	ITS	MT558602.1
BP484	*Fusarium sp.*	infected fruitlet	146	99,32	7,00E-68	ITS	MH777054.1
BP485	*Fusarium proliferatum*	healthy fruitlet	659	100	0	EF1α	MN539103.1
BP486	*Fusarium proliferatum*	infected fruitlet	660	100	0	EF1α	MK442093.1
BP487	*Phialemoniopsis curvata*	infected fruitlet	519	99,43	0	ITS	AB278180.1
BP488	*Fusarium verticillioides*	infected fruitlet	541	100	0	ITS	MT594370.1
BP490	*Phialemoniopsis curvata*	infected fruitlet	535	99,44	0	ITS	AB278180.1
BP492	*Fusarium verticillioides*	infected fruitlet	528	100	0	ITS	MT594370.1
BP493	*Phialemoniopsis curvata*	healthy fruitlet	508	99,41	0	ITS	AB278180.1
BP494	*Fusarium ananatum*	healthy fruitlet	661	100	0	EF1α	MT010996.1
BP495	*Phialemoniopsis curvata*	infected fruitlet	511	99,42	0	ITS	AB278180.1
BP496	*Phialemoniopsis curvata*	infected fruitlet	518	99,42	0	ITS	AB278180.1
BP497	*Fusarium oxysporum*	healthy fruitlet	510	100	0	ITS	EU152473.1
BP499	*Fusarium equiseti*	infected fruitlet	527	99,81	0	ITS	MH879584.1
BP500	*Fusarium ananatum*	infected fruitlet	659	100	0	EF1α	MT010996.1
BP501	*Phialemoniopsis curvata*	infected fruitlet	508	99,41	0	ITS	AB278180.1
BP503	*Phialemoniopsis curvata*	infected fruitlet	506	99,41	0	ITS	AB278180.1
BP504	*Phialemoniopsis curvata*	healthy fruitlet	507	99,21	0	ITS	AB278180.1
BP507	*Lasiodiplodia mahajangana*	healthy fruitlet	401	100	0	ITS	MH057188.1
BP511	*Fusarium verticillioides*	healthy fruitlet	660	100	0	EF1α	MN861780.1
BP512	*Fusarium verticillioides*	healthy fruitlet	660	100	0	EF1α	MN861772.1
BP513	*Fusarium proliferatum*	healthy fruitlet	671	100	0	EF1α	KF993985.1
BP515	*Aspergillus oryzae*	infected fruitlet	562	100	0	ITS	MN648727.1
BP516	*Aspergillus flavus*	infected fruitlet	515	100	0	ITS	MN955851.1
BP518	*Fusarium verticillioides*	healthy fruitlet	640	100	0	EF1α	MN861768.1
BP519	*Phialemoniopsis curvata*	healthy fruitlet	505	99,41	0	ITS	AB278180.1
BP520	*Clonostachys rosea*	healthy fruitlet	406	97,78	0	ITS	MH047188.1
BP521	*Phialemoniopsis curvata*	infected fruitlet	519	99,43	0	ITS	AB278180.1
BP522	*Fusarium verticillioides*	infected fruitlet	529	100	0	ITS	MT594370.1
BP523	*Fusarium proliferatum*	healthy fruitlet	656	99,7	0	EF1α	KU872092.1
BP526	*Fusarium dlamini*	healthy fruitlet	538	100	0	ITS	MN173109.1
BP527	*Fusarium proliferatum*	infected fruitlet	660	100	0	EF1α	KF993985.1
BP528	*Fusarium solani*	infected fruitlet	541	100	0	ITS	MT594367.1
BP529	*Fusarium verticillioides*	infected fruitlet	636	100	0	EF1α	MN861780.1
BP530	*Fusarium ananatum*	healthy fruitlet	656	100	0	EF1α	MT010996.1
BP532	*Fusarium solani*	infected fruitlet	541	100	0	ITS	MT594367.1

Table S1. Continued

Isolate ID	Identification	Sampled tissue	Fragment size (bp)	Identity (%)	E-value	Locus	GenBank accession number
BP533	*Fusarium verticillioides*	infected fruitlet	656	100	0	EF1α	MH496632.1
BP534	*Fusarium proliferatum*	infected fruitlet	660	100	0	EF1α	MK442093.1
BP536	*Fusarium verticillioides*	healthy fruitlet	658	100	0	EF1α	MT095057.1
BP537	*Phialemoniopsis curvata*	healthy fruitlet	389	99,49	0	ITS	AB278180.1
BP538	*Lasiodiplodia citricola*	healthy fruitlet	416	100	0	β-tubulin	KU530119.1
BP539	*Lasiodiplodia theobromae*	healthy fruitlet	415	100	0	β-tubulin	KR260829.1
BP540	*Fusarium sp.*	healthy fruitlet	514	100	0	ITS	FJ008991.1
BP543	*Fusarium fujikuroi*	healthy fruitlet	378	100	0	ITS	MN963682.1
BP544	*Fusarium proliferatum*	infected fruitlet	659	100	0	EF1α	MT095058.1
BP546	*Fusarium solani*	healthy fruitlet	535	100	0	ITS	JN006817.1
BP548	*Fusarium solani*	healthy fruitlet	548	100	0	ITS	KX621960.1
BP549	*Fusarium falciforme*	healthy fruitlet	538	100	0	ITS	MT251175.1
BP550	*Aspergillus flavus*	infected fruitlet	576	100	0	ITS	MF120213.1
BP551	*Aspergillus niger*	infected fruitlet	565	100	0	ITS	MN788116.1
BP555	*Fusarium sp.*	healthy fruitlet	610	99,02	0	EF1α	JF740861.1
BP556	*Fusarium proliferatum*	infected fruitlet	661	100	0	EF1α	MN386746.1
BP559	*Fusarium sp.*	healthy fruitlet	645	98,77	0	EF1α	JF740861.1
BP563	*Fusarium solani*	infected fruitlet	550	100	0	ITS	KX621960.1
BP564	*Fusarium proliferatum*	healthy fruitlet	661	100	0	EF1α	MN386746.1
BP565	*Fusarium ananatum*	healthy fruitlet	658	100	0	EF1α	MT010996.1
BP566	*Fusarium sp.*	infected fruitlet	516	100	0	ITS	MT530119.1
BP567	*Fusarium solani*	healthy fruitlet	543	100	0	ITS	MT594367.1
BP570	*Fusarium proliferatum*	healthy fruitlet	663	100	0	EF1α	KF993986.1
BP572	*Fusarium fujikuroi*	healthy fruitlet	452	100	0	ITS	MN963682.1
BP573	*Fusarium equiseti*	healthy fruitlet	495	100	0	ITS	MN589630.1
BP574	*Fusarium solani*	infected fruitlet	638	99,69	0	EF1α	KY486693.1
BP575	*Fusarium sacchari*	infected fruitlet	660	100	0	EF1α	MN193868.1
BP576	*Phialemoniopsis curvata*	healthy fruitlet	511	99,42	0	ITS	AB278180.1
BP578	*Phialemoniopsis curvata*	infected fruitlet	519	99,44	0	ITS	AB278180.1
BP579	*Fusarium chlamydosporum*	healthy fruitlet	270	100	1,00E-138	β-tubulin	KJ125830.1
BP580	*Aspergillus flavus*	infected fruitlet	548	100	0	ITS	MN955851.1
BP582	*Davidiella sp.*	healthy fruitlet	577	100	0	ITS	KX621979.1
BP583	*Aspergillus flavus*	healthy fruitlet	558	100	0	ITS	MN006669.1
BP584	*Aspergillus welwitschiae*	healthy fruitlet	560	100	0	ITS	MH374611.1
BP585	*Aspergillus flavus*	healthy fruitlet	242	100	0	ITS	MK850352.1
BP586	*Aspergillus flavus*	infected fruitlet	552	99,82	0	ITS	MN238861.1
BP589	*Talaromyces stollii*	infected fruitlet	405	100	0	β-tubulin	JX315634.1
BP590	*Fusarium sp.*	healthy fruitlet	651	98,79	0	EF1α	JF740861.1
BP594	*Penicillium sp.*	healthy fruitlet	496	100	0	ITS	KU556148.1
BP595	*Fusarium sp.*	healthy fruitlet	627	98,58	0	EF1α	JF740861.1
BP596	*Phialemoniopsis curvata*	infected fruitlet	518	99,43	0	ITS	AB278180.1
BP597	*Phialemoniopsis curvata*	healthy fruitlet	520	99,44	0	ITS	AB278180.1
BP598	*Phialemoniopsis curvata*	infected fruitlet	520	99,44	0	ITS	AB278180.1
BP599	*Fusarium fujikuroi*	healthy fruitlet	524	100	0	ITS	CP023090.1
BP601	*Phialemoniopsis curvata*	infected fruitlet	506	99,21	0	ITS	AB278180.1
BP603	*Talaromyces amestolkiae*	healthy fruitlet	403	100	0	β-tubulin	MH287203.1
BP604	*Talaromyces purpureogenus*	infected fruitlet	548	100	0	ITS	KJ528885.1
BP605	*Talaromyces amestolkiae*	infected fruitlet	402	100	0	β-tubulin	KJ413360.1
BP611	*Fusarium oxysporum*	infected fruitlet	665	100	0	EF1α	CP053267.1
BP613	*Fusarium verticillioides*	healthy fruitlet	659	100	0	EF1α	MN861780.1

Table S2. Identification of fungal species isolated from soil coring points of cultures proximal to pineapple plots.

Isolate ID	Identification	Adjacent crop	Fragment size (bp)	Identity (%)	E-value	GenBank accession number
SBP050	*Acrostalagmus sp.*	jackfruit trees	411	100	0	MH482850.1
SBP027	*Aspergillus flavipes*	mango orchard	569	99,82	0	KF624764.1
SBP035	*Aspergillus flavipes*	mango orchard	550	100	0	KF624764.1
SBP068	*Aspergillus flavipes*	mango orchard	576	100	0	KF624764.1
SBP002	*Aspergillus sclerotiorum*	mango orchard	588	100	0	KC478519.1
SBP010	*Aspergillus sp.*	mango orchard	410	100	0	MK312294.1
SBP005	*Aspergillus subramanianii*	mango orchard	483	100	0	MK952313.1
SBP044	*Aspergillus tardicrescens*	jackfruit trees	449	99,78	0	KY087686.1
SBP020	*Aureobasidium namibiae*	mango orchard	196	100	2,00E-96	MK794387.1
SBP071	*Bartalinia pondoensis*	mango orchard	470	100	0	NR153599.1
SBP060	*Bartalinia pondoensis*	jackfruit trees	564	100	0	NR153599.1
SBP040	*Cladosporium cladosporioides*	jackfruit trees	479	98,96	0	MF475948.1
SBP012	*Cladosporium sp.*	mango orchard	418	100	0	MK336600.1
SBP029	*Cladosporium sp.*	mango orchard	542	100	0	MK336600.1
SBP062	*Cladosporium sp.*	jackfruit trees	542	100	0	MN275867.1
SBP043	*Cladosporium tenuissimum*	jackfruit trees	547	99,63	0	KX621982.1
SBP083	*Cystofilobasidiales sp.*	jackfruit trees	36	97,22	1,00E-06	MF615083.1
SBP053	*Diaporthe helianthi*	jackfruit trees	556	100	0	KM979926.1
SBP065	*Diaporthe helianthi*	jackfruit trees	563	100	0	KM979926.1
SBP006	*Edenia gomezpompae*	mango orchard	526	99,81	0	NR156217.1
SBP022	*Fennellia nivea*	mango orchard	577	100	0	FJ155814.1
SBP069	*Fennellia nivea*	mango orchard	563	100	0	FJ155814.1
SBP017	*Fusarium cf. solani*	mango orchard	434	99,77	0	MK336488.1
SBP037	*Fusarium equiseti*	jackfruit trees	492	100	0	MG650603.1
SBP076	*Fusarium oxysporum*	mango orchard	412	100	0	MH777057.1
SBP051	*Fusarium polyphialidicum*	jackfruit trees	517	100	0	HQ607880.1
SBP054	*Fusarium sp.*	jackfruit trees	508	100	0	KM099504.1
SBP064	*Fusarium sp.*	jackfruit trees	396	100	0	KM099504.1
SBP023	*Heterocephalum aurantiacum*	mango orchard	374	99,73	0	MH858803.1
SBP079	*Heterocephalum aurantiacum*	mango orchard	419	100	0	MH858803.1
SBP021	*Lecanicillium saksenae*	mango orchard	565	100	0	AB360363.1
SBP034	*Lecanicillium saksenae*	mango orchard	537	100	0	AB360363.1
SBP073	*Lecanicillium saksenae*	mango orchard	543	100	0	KY320616.1
SBP028	*Leptosphaeria spegazzinii*	mango orchard	524	99,81	0	MG664743.1
SBP038	*Mortierella horticola*	jackfruit trees	350	99,71	6,00E-180	KC018245.1
SBP007	*Mortierella sp.*	mango orchard	515	95,34	0	KU612370.1
SBP004	*Penicillium citrinum*	mango orchard	410	100	0	KX363454.1
SBP009	*Penicillium guanacastense*	mango orchard	523	99,24	0	MH374548.1
SBP052	*Penicillium janthinellum*	jackfruit trees	444	100	0	MG845261.1
SBP067	*Penicillium multicolor*	mango orchard	521	100	0	MH864874.1
SBP003	*Penicillium ochrochloron*	mango orchard	555	99,82	0	MG661735.1
SBP019	*Penicillium sclerotigenum*	mango orchard	561	100	0	MK334370.1
SBP011	*Penicillium sp.*	mango orchard	564	100	0	KT336528.1
SBP018	*Penicillium sp.*	mango orchard	556	100	0	HQ608086.1
SBP026	*Penicillium spinulosum*	mango orchard	565	99,65	0	MF476047.1
SBP039	*Penicillium sumatraense*	jackfruit trees	529	100	0	MH864547.1
SBP041	*Penicillium sumatraense*	jackfruit trees	558	100	0	MH864547.1
SBP045	*Penicillium sumatraense*	jackfruit trees	529	100	0	MH864547.1
SBP072	*Peniophora sp.*	mango orchard	826	100	0	HQ608067.1
SBP056	*Pestalotiopsis sp.*	jackfruit trees	267	100	0	LC040892.1
SBP049	*Phoma sp.*	jackfruit trees	413	100	0	JN207265.1
SBP059	*Plectosphaerella cucumerina*	jackfruit trees	516	100	0	AB469880.1
SBP063	*Pleosporales sp.*	jackfruit trees	517	100	0	MK247832.1
SBP008	*Purpureocillium lilacinum*	mango orchard	582	99,66	0	LN809016.1
SBP058	*Pyrenochaeta sp.*	jackfruit trees	503	99,6	0	EU750693.1
SBP025	*Robillarda sessilis*	mango orchard	553	99,46	0	KR873255.1
SBP030	*Robillarda sessilis*	mango orchard	568	99,47	0	KR873255.1
SBP057	*Roussoella siamensis*	jackfruit trees	478	100	0	NR155716.1
SBP075	*Talaromyces purpurogenus*	mango orchard	536	100	0	AB872822.1
SBP055	*Trichoderma guizhouense*	jackfruit trees	293	100	4,00E-150	MN170570.1
SBP001	*Trichoderma harzianum*	mango orchard	440	100	0	MN326481.1
SBP066	*Xepicula leucotricha*	jackfruit trees	569	100	0	MH858391.1

Table S3. Identification of fungal species from DNA extracted from DGGE gels corresponding to *Fusarium*- and *Talaromyces*-inoculated pineapple fruitlets.

Band	Inoculated species	Identification	Identity (%)	E-value	GenBank accession number
a	*Fusarium sacchari*	*Fusarium sp.*	100	2×10^{-33}	FJ008992.1
b	*Fusarium ananatum*	*Fusarium sp.*	99.51	4×10^{-100}	FJ210605.1
c	*Talaromyces stollii*	*Talaromyces stollii*	99.58	9×10^{-117}	AB910938.1
d	*Talaromyces amestolkiae*	*Talaromyces amestolkiae*	100	1×10^{-104}	MT441607.1
